# Comprehensive Investigation on Associations between Dietary Intake and Blood Levels of Fatty Acids and Colorectal Cancer Risk

**DOI:** 10.3390/nu15030730

**Published:** 2023-02-01

**Authors:** Ying Lu, Doudou Li, Lijuan Wang, Han Zhang, Fangyuan Jiang, Rongqi Zhang, Liying Xu, Nan Yang, Shuhui Dai, Xiaolin Xu, Evropi Theodoratou, Xue Li

**Affiliations:** 1 The Key Laboratory of Intelligent Preventive Medicine of Zhejiang Province, Department of Big Data in Health Science, School of Public Health, Centre of Clinical Big Data and Analytics of The Second Affiliated Hospital, Zhejiang University School of Medicine, Hangzhou 310058, China; 2 College of Public Health, Zhengzhou University, Zhengzhou 450001, China; 3 Centre for Global Health, Usher Institute, University of Edinburgh, Edinburgh EH8 9AG, UK; 4 Cancer Research UK Edinburgh Centre, Medical Research Council Institute of Genetics and Cancer, University of Edinburgh, Edinburgh EH4 2XU, UK; 5 The Key Laboratory of Intelligent Preventive Medicine of Zhejiang Province, Hangzhou 310058, China

**Keywords:** meta-analyses, Fas, trans-FA, PUFA, MUFA, CRC

## Abstract

Background: Increasingly, studies have discovered that different fatty acids (Fas) are linked to colorectal cancer (CRC) risk. Methods: We systematically searched Embase and Medline databases to identify eligible studies that examined the associations of different types of Fas with CRC risk. The effect estimates and their 95% confidence intervals (Cis) were pooled using a random-effects model. Subgroup and sensitivity analyses were performed to examine the robustness of the study findings. Results: This study evaluated the associations of 28 dietary and 18 blood Fas with CRC risk by summarizing the most updated evidence from 54 observational and four Mendelian Randomization (MR) studies. The present findings suggested that high dietary intake of eicosapentaenoic acid (EPA), docosahexanoic acid (DHA), and docosapentaenoic acid (DPA) are related to low risk of CRC, while the *n*-6/*n*-3 PUFA ratio and trans-FA are related to high risk of CRC. The summary of all cohort studies found that a high intake of SFA and DHA was a protective factor for CRC, and a high intake of the *n*-6/*n*-3 PUFA ratio was a risk factor for CRC. In the subgroup analysis of cancer subsites, we found that the dietary intake of linoleic acid (LA) and trans-FA are risk factors, while DPA is a protective factor for colon cancer. High dietary DHA intake was associated with a lower risk of rectal cancer, while the dietary *n*-6/*n*-3 PUFA ratio was associated with a higher risk of rectal cancer. Meta-analysis of blood FA levels showed a significant reverse association between blood pentadecanoic acid and CRC risk, whilst other blood Fas showed no significant association with CRC risk. All included MR studies showed that high plasma arachidonic acid (AA) is associated with increased CRC risk. Conclusions: Current evidence on the dietary intake and blood levels of Fas in relation to CRC risk is less consistent. Future studies are needed to investigate how the metabolism of Fas contributes to CRC development.

## 1. Introduction

Colorectal cancer (CRC) is the third most commonly diagnosed cancer and the second leading cause of cancer-related deaths in the world [1]. Worldwide, about 1.9 million CRC cases have been diagnosed, and an estimated 0.9 million CRC-related deaths occurred in 2020 [1]. Studies suggested that the carcinogenesis and progression of CRC might be influenced by multiple factors, including environmental, behavioral and metabolic risks [2]. Epidemiological and animal studies have also demonstrated that dietary nutrients play a significant role in the pathogenesis of CRC [3,4,5,6].

Foregoing studies have shown a link between dietary and blood-derived Fas and CRC [7,8,9,10]. Nevertheless, the measurement and evaluation methods of Fas from different sources vary, so the accuracy of the data obtained varies [11,12,13]. Dietary intake of Fas is commonly measured by self-reported food frequency questionnaires, 24-h dietary recalls, or dietary records, which might lead to an inaccurate assessment of Fas intake [11,12]. Measurement of blood levels of Fas may provide a more precise assessment, but it usually does not have direct indications of dietary intake [13]. With these pros and cons, compiling evidence for the association with both dietary intake and blood levels of Fas is indispensable to explain their roles in the development of CRC.

A multitude of published meta-analysis studies found that various sources of Fas are associated with CRC [7,10,14,15,16,17,18,19,20,21], but not consistently. The most updated meta-analysis of 26 prospective cohorts explored the association between dietary Fas and CRC and found that high dietary intake of docosahexanoic acid (DHA) and eicosapentaenoic acid (EPA) and low dietary intake of linoleic acid (LA) intake were associated with a reduced risk of CRC [7]. Additionally, a pooled analysis of trans-FA published recently suggested that trans-FA was a risk factor for CRC [21]. However, the result from pooled studies showed that both dietary and blood-derived *n*-6 PUFA and CRC risk were not relevant [18,20]. The most recent meta-analysis of blood *n*-3 PUFA levels found an inverse association with CRC risk, and a 1% increase in blood *n*-3 was associated with a 4% decrease in CRC risk; this study did not examine the heterogeneous associations between specific types of *n*-3 PUFAs and CRC risk [10]. There is a lack of meta-analysis to synthesize evidence for both dietary and blood Fas in their relationships with CRC risk and to compare the evidence from MR studies to strengthen the causal inference.

In this study, we performed a meta-analysis of observational studies to assess the associations of both dietary intake and blood levels of Fas with the risk of CRC. In addition, we reviewed previously published MR studies to explore the causal association between plasma Fas and CRC risk based on genetic evidence. Identifying Fas that are associated with CRC risk with high-grade evidence would be beneficial to the formulation of dietary recommendations for CRC prevention.

## 2. Methods

### 2.1. Literature Search and Selection

We systematically searched Embase and Medline databases from inception to 13 January 2023 using a search strategy (Supplementary Table S1) to identify observational studies and MR studies that evaluated the association between specific Fas and the risk of CRC. Additionally, we reviewed the reference lists of retrieved articles and related articles in a published meta-analysis to identify potentially relevant articles. The literature screening was conducted independently by two investigators (YL and DDL). The protocol of the current study has been registered in the International prospective register of systematic reviews (PROSPERO) with the identification number CRD42022319949.

Studies were included in this meta-analysis when they met the following inclusion criteria: (1) observational studies (cohort study, case-control study, or nested case-control study) or MR studies; (2) the exposure was dietary Fas intake or blood levels of Fas; (3) the outcome was CRC, colon cancer or rectum cancer; (4) reported the effect estimates (odds ratio (OR), risk ratio (RR) or hazard ratio (HR)) and 95% confidence interval (CI). When more than one study was from the same cohort, we included the study with the largest population and longest follow-up period.

### 2.2. Data Extraction and Quality Assessment

Two investigators (YL and DDL) extracted data independently in accordance with the Preferred Reporting Items for Systematic Reviews and Meta-Analyses (PRISMA) statement [22]. Any discrepancies were addressed by checking the original studies and through discussion. The information extracted from each study is as follows: the lead author’s name, year of publication, the study design, sex of participants, number of participants, types of Fas, anatomical subsites of the tumor (colon, rectum, colorectal), and the adjusted effect estimates (OR, HR, RR) with 95% Cis. Two investigators (YL and DDL) extracted data independently using a predesigned data extraction form. The Newcastle-Ottawa quality assessment scale (NOS) was applied to evaluate the quality of the included observational studies [23]. The checklist contains three quality parameters: selected population, comparability of groups, and evaluation of exposures (for case-control) or outcomes (for cohorts) of interest [23]. Each study was scored on a scale of 0 to 9. Studies with scores greater than or equal to 7 are generally considered to be of high quality [23]. The quality of each MR study was assessed independently by two investigators (YL and DDL) based on accepted criteria as previously reported [24]. The robustness of evidence is divided into four categories (robust, probable, suggestive, insufficient evidence), based on the consistency of the findings between different MR analytical methods The detailed scoring details for this method have been described in detail in previous articles [24].

### 2.3. Statistical Analysis

We performed meta-analyses of observational studies using the DerSimonian and Laird random-effects model [25], which considered both within- and between-study variation. In addition, we performed stratified analyses by the study design to examine the influence of the study design on the results, and we also performed subgroup meta-analyses to examine the association between Fas and different cancer subsites. The Cochran Q statistic and I^2^ metric were used to test the statistical heterogeneity and inconsistencies among studies [26,27], I^2^ values of 25%, 50%, and 75% corresponded to cutoff points for low, moderate, and high degrees of heterogeneity. Begg’s and Egger’s tests were used to assess potential publication bias [28]. Bonferroni correction was applied to account for the multiple testing (a total of 28 FAs), where *p*-value < 0.002 (0.05/28) was regarded as statistically significant and *p*-value < 0.05 was regarded as nominally significant [29]. All statistical analyses were performed using the “metafor” and “forestplot” R packages, R software version 4.0.2 (The R Foundation, Boston, MA, USA). Findings from MR studies were synthesized narratively and thematically to complement and strengthen the causal inference for the associations reported by observational studies.

## 3. Results

### 3.1. Meta-Analyses of Observational Studies

**Figure 1:**
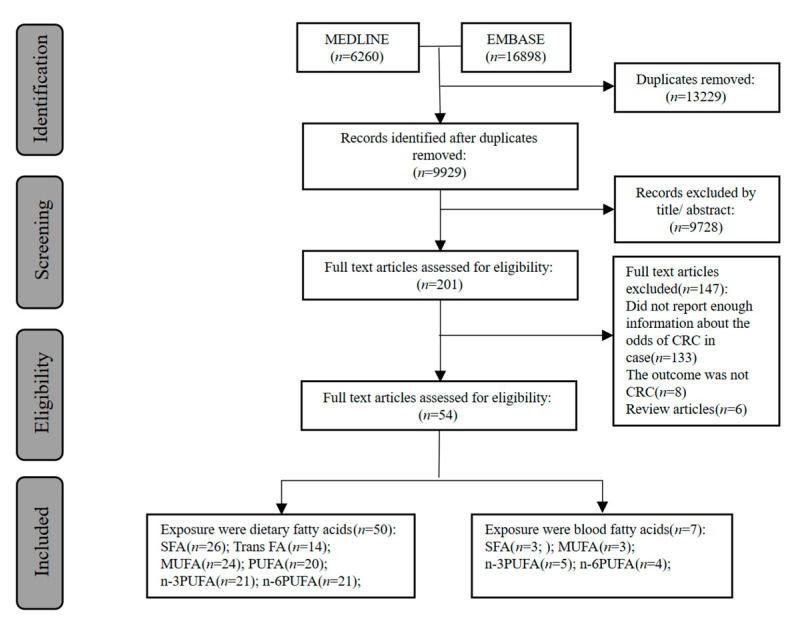
Flow diagram of observational and Mendelian randomization studies selection.

As shown in Figure 1, our search identified 9929 potentially relevant studies. After screening the title and abstracts, 201 studies were left for reviewing the full text, and 54 original studies including 52,980 cases among 2,496,950 participants were included in the final meta-analyses. All included studies were published between 1980 and 2022, including 29 prospective studies, 23 case-control, and two nested case-control or case-cohort studies. According to the NOS checklist, 42 studies were of high quality (score ≥ 7). (Supplementary Tables S2 and S3). Supplementary Tables S4 and S5 summarized the main information of the included studies. Some studies examined the association of overall SFA, trans-FA, MUFA, and PUFA with CRC, while others considered specific types of these FAs groups. The information on FAs intake was mainly obtained from food frequency questionnaires (*n* = 41), diet history questionnaires (*n* = 6), food diaries (*n* = 2), and 24-h diet recall (*n* = 2). Blood FAs data were obtained from gas chromatography (*n* = 5) and gas-liquid chromatography (*n* = 2). The features of Mendelian randomization studies of plasma FAs and CRC were included in Supplementary Table S6.

In summary, 50 studies that included 52,980 cases and 2,495,256 participants evaluated the association between dietary FAs and CRC, including SFA, trans-FA, MUFA, PUFA, *n*-3 PUFA, *n*-6 PUFA, *n*-6/*n*-3 PUFA ratio, and *n*-3/*n*-6 PUFA ratio; seven studies with 2086 cases and 7570 participants examined the relationship between blood FAs and CRC, including SFA, MUFA, *n*-3 PUFA, *n*-6 PUFA, and *n*-6/*n*-3 PUFA ratio. Supplementary Table S6 showed the types of FAs included in MR studies of plasma FAs and CRC.

### 3.2. Association between Dietary Fatty Acids Intake and Colorectal Cancer

#### 3.2.1. Main Analysis

**Figure 2:**
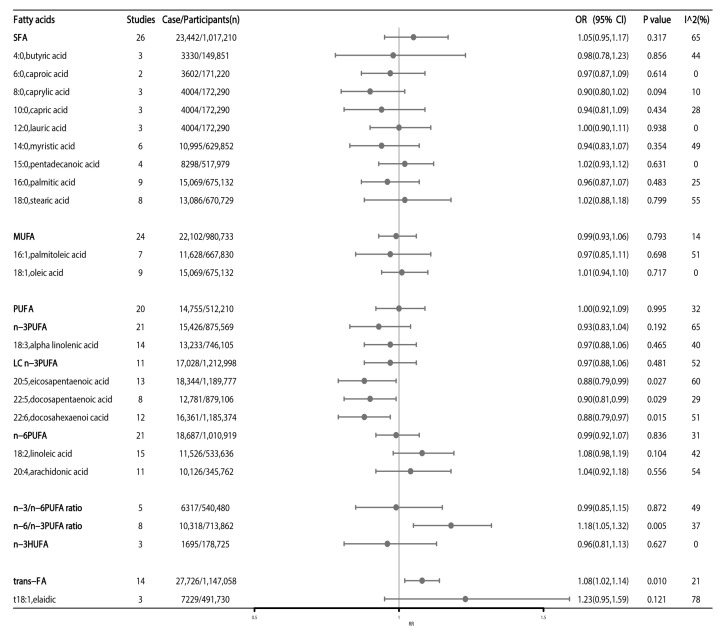
High versus low fatty acids intake and association with colorectal cancer risk. Estimates are odds ratio and meta-analyses are based on random effect models. SFA: saturated fatty acids; MUFA: monounsaturated fatty acids; PUFA: polyunsaturated fatty acids; HUFA: highly unsaturated fatty acids; trans-FA: trans-fatty acids; LC *n*-3 fatty acids: long-chain *n*-3 fatty acids.

Our meta-analyses examined the associations between SFA, MUFA, PUFA, *n*-3 PUFA, long-chain *n*-3 PUFA (LC *n*-3 PUFA), highly unsaturated fatty acids (*n*-3 HUFA), *n*-6 PUFA, *n*-3/*n*-6 PUFA ratio intake and CRC risk (Figure 2). Higher intake of trans-FA and higher *n*-6/*n*-3 PUFA ratio was associated with an increased CRC risk, the pooled OR being 1.08 (95%CI: 1.02–1.14, *p* = 0.010) and 1.18 (95%CI: 1.05–1.32, *p* = 0.005), respectively (Figure 2).

All types of SFA (including butyric acid, caproic acid, caprylic acid, capric acid, lauric acid, pentadecanoic acid, palmitic acid, myristic acid, and stearic acid) had no impact on the risk of CRC (Figure 2). Regarding the type of MUFA, both palmitoleic acid and oleic acid had no impact on the risk of CRC (Figure 2). For *n*-3 PUFA, intake of LC *n*-3 PUFA and ALA was not significantly associated with CRC risk (Figure 2), while intake of other types of *n*-3 PUFA, including EPA, DPA, and DHA, was associated with 12% (OR:0.88; 95%CI: 0.79–0.99, *p* = 0.027), 10% (OR:0.90, 95%CI: 0.81–0.99, *p* = 0.029) and 12% (OR:0.88; 95%CI: 0.79–0.97, *p* = 0.015) lower risk of CRC, respectively (Figure 2). Finally, the two main types of dietary *n*-6 PUFA (LA and AA) and elaidic showed no significant associations with CRC risk (Figure 2).

#### 3.2.2. Subgroup Analysis

In the subgroup analysis of cancer sites, dietary DPA, LA, and trans-FA FAs were nominally associated with colon cancer and the summary ORs were 0.87 (95%CI: 0.76–0.99; *p* = 0.040), 1.15 (95%CI: 1.02–1.29; *p* = 0.023), and 1.14 (95%CI: 1.02–1.27; *p* = 0.019), respectively (Table 1). Dietary DHA and *n*-6/*n*-3 PUFA ratios were nominally associated with rectal cancer risk, and the summary ORs were 0.87 (95% CI: 0.76–0.99; *p* = 0.041) and 1.25 (95% CI: 1.05–1.48; *p* = 0.013), respectively (Table 1).

**Figure 3:**
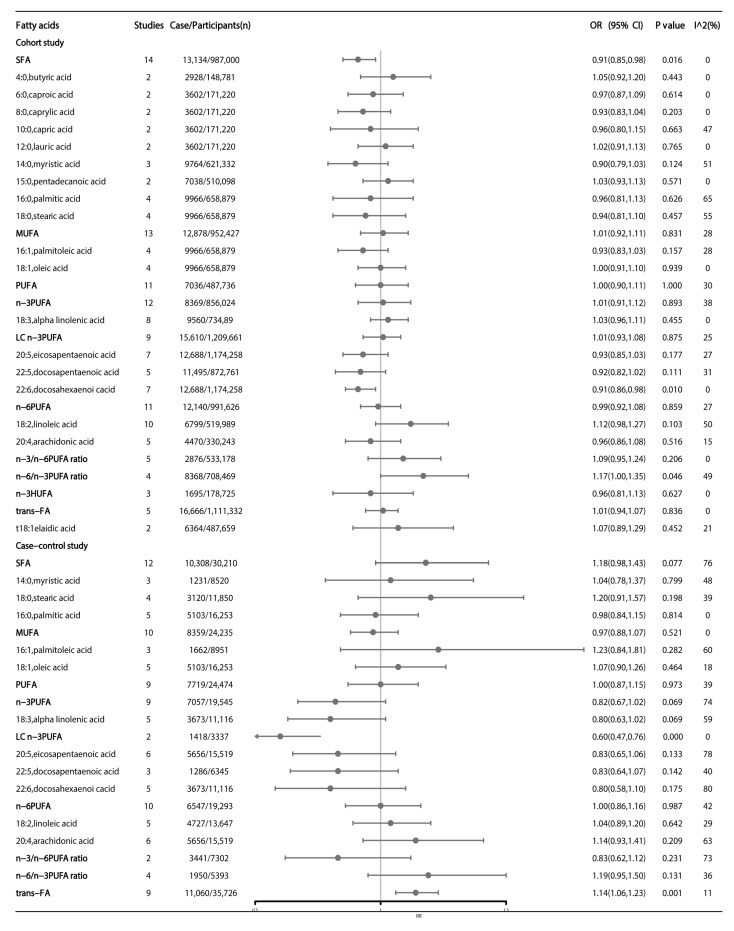
Subgroup analysis of dietary fatty acids and colorectal cancer by the study design. Estimates are odds ratio and meta-analyses are based on random effect models. SFA: saturated fatty acids; MUFA: monounsaturated fatty acids; PUFA: polyunsaturated fatty acids; HUFA: highly unsaturated fatty acids; trans-FA: trans-fatty acids; LC *n*-3 fatty acids: long-chain *n*-3 fatty acids.

In subgroup analyses of study design, results from pooled cohort studies showed that high dietary DHA and *n*-6/*n*-3 PUFA ratios were still nominally associated with CRC, and the summary OR was 0.91 (95% CI: 0.86–0.98; *p* = 0.010) and 1.17 (95% CI: 1.00–1.35; *p* = 0.046), respectively. Additionally, high SFA intake was found to be a protective factor for CRC, the summary OR was 0.91 (95% CI: 0.85–0.98; *p* = 0.016) (Figure 3). Results from pooled case-control studies showed that high dietary intake of LC *n*-3 PUFA was significantly associated with reduced CRC risk (OR = 0.60; 95% CI: 0.47–0.76; *p* < 0.001), whilst high trans-FA was nominally associated with high CRC risk (OR = 1.14; 95% CI: 1.06–1.23; *p* = 0.001) (Figure 3).

#### 3.2.3. Association between Blood Fatty Acids and Colorectal Cancer

**Figure 4:**
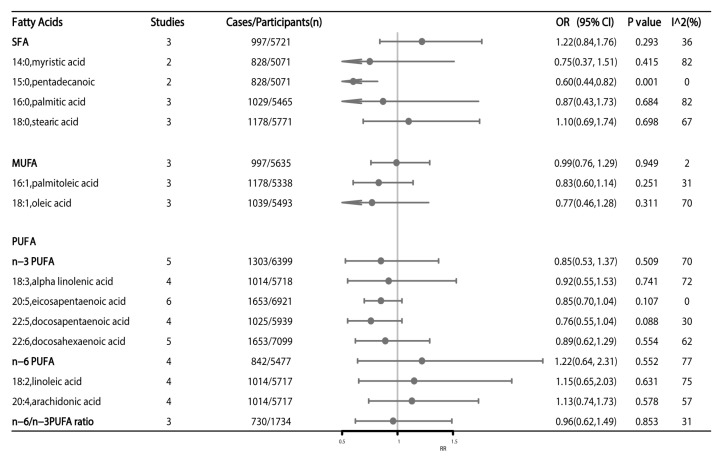
High versus low level of blood fatty acids and association with colorectal cancer risk. Estimates are odds ratio and meta-analyses are based on random effect models. SFA: saturated fatty acids; MUFA: monounsaturated fatty acids; PUFA: polyunsaturated fatty acids.

A total of seven articles have reported the association between blood FAs and CRC risk, of which a meta-analysis of two articles showed a significant reverse association between blood pentadecanoic acid (RR: 0.60; 95%CI: 0.44–0.82, *p* = 0.001) and CRC risk (Figure 4). Other blood FAs showed no significant association with CRC risk (Figure 4).

#### 3.2.4. Mendelian Randomization Studies

As shown in Figure 1, we identified four MR studies assessing the causal effect of FAs on CRC risk, of which three MR studies were performed among Europeans, and one MR included both European and Asian populations. Among the four MR analyses included, the evidence grading results were: robust evidence (*n* = 1), suggestive evidence (*n* = 2), and non-evaluable evidence (*n* = 1). The eligible MR studies assessed the following 12 exposures: arachidic acid, palmitic acid (PA), stearic acid (SA), DHA, DPA, EPA, AA, dihomo-g-linolenic acid (DGLA), LA, POA, OA, and ALA. Six FAs were examined by more than one MR study. All included MR studies have shown that high plasma AA, SA, DPA, and EPA were associated with increased CRC risk, whilst high plasma LA, OA, POA, and ALA were related to a decreased risk of CRC. Other FAs reported in these MR studies, including arachidic acid, PA, DHA, and DGLA, showed no significant association with CRC risk (Supplementary Table S6).

## 4. Discussion

This study evaluated the associations of dietary and blood FAs with the risk of CRC by summarizing the most updated evidence from 54 observational studies and four MR studies. The present findings suggest that a high dietary intake of EPA, DHA, and DPA, is related to a low risk of CRC, while the *n*-6/*n*-3 PUFA ratio and trans-FA are related to a high risk of CRC. The association between dietary DHA, the *n*-6/*n*-3PUFA ratio, and trans-FA intake and CRC risk were validated by study-type subgroup analyses. In terms of cancer subsites, we found that high dietary intake of LA and trans-FA are risk factors for colon cancer, while high dietary intake of DPA is a protective factor for colon cancer. The dietary *n*-6/*n*-3PUFA ratio is a risk factor for rectal cancer, while DHA is a protective factor for rectal cancer. Meta-analysis of blood FA levels showed a significant reverse association between blood pentadecanoic acid and CRC risk, whilst other blood FAs showed no significant association with CRC risk. All included MR studies showed that high plasma AA is associated with increased CRC risk. Overall, current evidence on the dietary intake and blood levels of FAs in relation to CRC risk is less consistent.

### 4.1. N-3 Polyunsaturated Fatty Acids (n-3 PUFA)

Our meta-analysis suggested that high dietary intake of DHA (low heterogeneity), DPA (moderate heterogeneity), and EPA (moderate heterogeneity), were associated with reduced risk of CRC. Although the results of most observational studies have shown no meaningful association between *n*-3 PUFA and CRC, there has been an inverse association between dietary EPA, DHA, and DPA intake and CRC in previously published studies [7,10,30,31]. Nevertheless, no significant association between blood *n*-3 PUFA and CRC was observed, which could be due to relatively small sample sizes and variations of the main sources of blood *n*-3 PUFA which are abundant in fish and fish oil [32]. Former studies suggested that the protective effect of *n*-3 PUFA against CRC is due to the intake of refined fish oils rich in EPA and DHA [33]. There is evidence indicating that higher levels of *n*-3 PUFA can regulate inflammatory mediators and immune cell function in the tumor microenvironment [34], showing a significant association with a decreased risk of CRC [35,36]. The intake of EPA + DHA varies with the dietary patterns of different populations; For example, fish and fatty fish intake is higher in the Japanese population but lower in most Western diets, which may also influence the link between these FAs and CRC [37]. However, evidence from published MR studies is quite conflicting, which indicated that high plasma DPA was associated with an increased risk of CRC. Taken together, the association between *n*-3 PUFA and CRC risk needs to be confirmed with more high-quality prospective cohort studies.

### 4.2. N-6 Polyunsaturated Fatty Acids (n-6 PUFA)

Our meta-analysis showed that the dietary *n*-6/*n*-3 PUFA ratio and high intake of *n*-6 PUFA (i.e., LA) were associated with an increased risk of CRC. Not only have cohort studies shown that the dietary *n*-6/*n*-3 PUFA ratio is a risk factor for CRC [30], but a randomized controlled trial also found that the ratio of *n*-6/*n*-3 PUFA was significantly higher in tumor versus non-tumor tissue [38]. The latest meta-analysis of 26 cohort studies has found that individuals with high dietary intake of LA had an increased risk of CRC [7], which was in line with our findings. LA is an *n*-6 PUFA found mainly in vegetable oils such as soybean, corn, and safflower [39]. LA is the most commonly used dietary vegetable oil in the United States. Although the recommended ratio of *n*-6/*n*-3 PUFAs is 1:1 to 2:1, in a typical Western diet, the ratio of *n*-6/*n*-3 PUFAs is much larger than recommended [39]. High *n*-6 PUFA diets induce bile excretion of bile acid, increase the level of secondary bile acid deoxycholic acid in feces, and chemically induce colon cancer [40]. Epigenetic analysis showed that long-term exposure to high *n*-6 PUFA diets down-regulated APC expression through CpG hypermethylation, and was associated with increased COX-2 expression through PTSC-2 CpG hypomethylation, which increased the risk of inflammation and colon cancer [41]. Due to the antithetical relationship between *n*-6 and *n*-3 PUFA in the endosomal inflammatory response, a balanced intake of *n*-6 and *n*-3 PUFA should be recommended for the prevention of CRC.

### 4.3. Saturated Fatty Acids (SFAs)

We found an inverse association between SFA intake and CRC risk in our pooled cohort study results, which was consistent with findings from a large nutrition cohort study published in 2021 [8]. SFA can be divided into odd-chain fatty acids (OCFA) and even-chain fatty acids (ECFA) based on the number of carbon atoms. Meta-analysis results showed that blood-derived pentadecanoic acid (OCFA) was significantly associated with a reduced risk of CRC. Dietary and plasma SFAs are strongly associated with high-fat dairy foods [42], and studies have found negative associations between SFA levels and many diseases [43,44]. Pentadecanoic acid was thought to be mainly derived from dairy products and had been shown to have a positive association with health which is related to several diseases [45]. We were unable to demonstrate a firm conclusion that blood pentadecanoic acid was associated with CRC risk due to limited research; more studies are needed to dissect this observed association.

### 4.4. Trans Fatty Acids (Trans-FA)

Trans-FA from different dietary sources, including industrial fatty acids (iTFA) and ruminant fatty acids (rTFA), have different activities. Previous studies that investigated dietary intakes of total trans-FA iTFA and rTFA intake and CRC risk have been inconclusive [11,46,47,48,49]. However, both current and previous evidence consistently support that a high intake of dietary trans-FA is associated with an increased risk of CRC, particularly colon cancer [46]. A recent pooled meta-analysis also reported the association between trans-FA and CRC [21]. In a prospective study of the Women’s Health Survey, a significant positive association was found between fried food consumption and CRC risk [49], which supports our findings on a dietary level. Results from previous meta-analyses suggested that high consumption of trans-FA is potentially harmful in the context of common cancers and supports the prohibition of industrial trans-FA in foods [21]. Studies have shown that a high intake of trans-FA was associated with the development of inflammation and that elevated levels of inflammatory biomarkers in the blood caused damage to genes and protein molecules that promote cancer cell proliferation [50,51]. However, given that epidemiological studies on the association between trans-FA and CRC have primarily been conducted through dietary assessments, future studies need to include multiple sources of trans-FA to minimize errors in dietary intake assessments.

### 4.5. Conflict and Harmony of Dietary and Blood FAs in Their Associations with CRC

Meta-analysis showed that high dietary intake of LA was associated with increased colon cancer, while genetic studies showed the opposite direction of association between blood LA and CRC and there was strong evidence showing that high plasma arachidonic acid (AA), a membrane phospholipid originated from LA, was associated with increased CRC risk at the genetic level [52,53]. In the UK, the majority (at least 90%) of adults’ *n*-6 PUFA intake is LA [54], which means that energy from *n*-6 PUFA is mainly from LA. Moreover, dietary *n*-6 PUFA has been discussed above as a risk factor for CRC. These complex associations might be explained by the action of desaturases through which the dietary intake of LA could be rapidly converted into circulating AA [55,56]. In other words, a high dietary intake of LA would result in a high blood level of AA. Moreover, it has been elucidated that the rate-limiting step of AA production from LA is the initial desaturation of linoleic acid via delta-6 desaturase, and the inhibition of this desaturation encumbers tumorigenesis in mouse models [57]. It has been known that high blood AA would result in excessive production of proinflammatory prostaglandin E2 (PGE2) through the cycloxygenase pathway, which might be the culprit contributing to the development of CRC [58].

### 4.6. Study Strengths and Limitations

To the best of our knowledge, this is the first comprehensive meta-analysis to investigate the risk of CRC in relation to both dietary intake and blood levels of FAs. Since dietary FAs intake was assessed mainly by a food frequency questionnaire (FFQ) or dietary diary, which may be subject to recall bias and information bias, we also pooled blood FAs data to systematically assess the association between FAs from different sources and CRC. Meanwhile, we included MR studies to review the causal relationship between plasma FAs levels and CRC at the genetic level to integrate directionally consistent associations between multiple FAs and CRC risk. However, some limitations should also be considered while interpreting the present findings. Firstly, the dietary intake of FAs was collected by a questionnaire which might present recall bias. Secondly, the meta-analysis observed heterogeneity between studies, for which we performed a series of subgroup analyses to minimize the sources of heterogeneity. Thirdly, we did not conduct a dose-response analysis due to the lack of data, and therefore, were not able to examine the non-linearity of their associations. Finally, we were unable to conclude the relationships between different dietary sources of FAs and CRC risk due to the limited information about the dietary sources of specific FAs reported in the original articles.

## 5. Conclusions

In conclusion, the present study concluded that the high dietary intake of DHA, DPA, and EPA, and high blood levels of pentadecanoic acid are protective factors for CRC, and the summary ORs were 0.88 (95% CI: 0.79–0.97; *p* = 0.015), 0.90 (95% CI: 0.81–0.99; *p* = 0.029), 0.88 (95% CI: 0.79–0.99; *p* = 0.027) and 0.60 (95% CI: 0.44–0.82; *p* = 0.001), respectively; while high dietary derived *n*-6/*n*-3 PUFA ratios and trans-FA were risk factors for CRC, and the summary ORs were 1.18 (95% CI: 1.05–1.32; *p* = 0.005) and 1.08 (95% CI: 1.02–1.14; *p* = 0.010). Findings from our study provide the most updated and comprehensive evidence on dietary FAs intake for colorectal cancer. Identifying FAs associated with CRC risk with a high level of evidence will help develop dietary recommendations to prevent CRC. Clinical trials of diet-related intervention in accompaniment with immunotherapy to improve the prognosis of CRC may be worth consideration.

## Figures and Tables

**Table 1:** Subgroup analysis of dietary fatty acids and risk of colon and rectal cancer.

Fatty Acid	Colon Cancer					Rectum Cancer				
	No of Studies	Cases/Total Participants	RR (95% CI)	*p* Value	I^2^ (%)	No of Studies	Cases/Total Participants	RR (95% CI)	*p* Value	I^2^ (%)
***n*-3 PUFA**	7	4268/310,898	0.83 (0.67, 1.02)	0.077	59	7	1786/314,886	1.02 (0.80, 1.29)	0.873	38
ALA	6	2716/315,918	0.96 (0.83, 1.11)	0.597	0	6	2461/396,911	1.02 (0.83, 1.27)	0.824	46
EPA	5	7591/734,064	0.93 (0.82, 1.06)	0.264	24	4	2873/729,661	0.87 (0.72, 1.04)	0.133	12
DHA	5	6148/789,647	0.92 (0.81, 1.04)	0.174	15	5	3215/789,647	0.87 (0.76, 0.99)	0.041	0
DPA	4	5686/672,953	0.87 (0.76, 0.99)	0.040	16	4	2966/672,953	0.90 (0.78, 1.03)	0.132	0
***n*-6 PUFA**	6	2253/272,456	0.96 (0.83, 1.13)	0.645	0	8	2976/397,186	1.03 (0.82, 1.28)	0.824	43
LA	7	3950/281,344	1.15 (1.02, 1.29)	0.023	0	6	1170/248,618	1.19 (0.89, 1.61)	0.246	55
AA	4	2918/185,865	0.98 (0.84, 1.14)	0.799	0	3	607/181,462	0.81 (0.63, 1.04)	0.100	0
**Total PUFA**	8	4660/141,264	1.07 (0.97, 1.19)	0.165	10	4	1060/70,044	1.02 (0.86, 1.22)	0.811	23
**Total MUFA**	11	5407/239,203	0.97 (0.87, 1.07)	0.517	12	7	1728/80,303	1.01 (0.88, 1.16)	0.892	0
Oleic acid	3	2951/70,043	0.88 (0.76, 1.03)	0.105	4	-	-	-	-	-
**Total SFA**	10	6371/233,272	0.99 (0.89, 1.10)	0.844	7	6	1605/72,358	1.01 (0.87, 1.17)	0.876	3
Palmitic acid	3	2951/68,029	0.91 (0.77, 1.07)	0.242	0	-	-	-	-	-
**Trans FA**	3	3087/9692	1.14 (1.02, 1.27)	0.019	10	-	-	-	-	-
**Ratio *n*-6/*n*-3**	-	-	-	-	-	2	2226/549,403	1.25 (1.05, 1.48)	0.013	0

1OR: odds ratio. 2SFA: saturated fatty acids; MUFA: monounsaturated fatty acids; PUFA: polyunsaturated fatty acids; trans FA: trans fatty acids.

## Data Availability

Data described in the manuscript, code book, and analytic code will be made available upon request pending application and approval.

## Supplementary Materials

The following supporting information can be downloaded at: https://www.mdpi.com/article/10.3390/nu15030730/s1, Table S1. Search strategy. Table S2. Results of quality assessment obtained with the Newcastle-Ottawa Scale for cohort studies. Table S3. Results of quality assessment obtained with the Newcastle-Ottawa Scale for case control studies. Table S4. Characteristics of the included studies in the meta-analysis of the intake of fatty acids and colorectal cancer. Table S5. Characteristics of the included studies in the meta-analysis. Table S6. Characteristics of the included Mendelian Randomization studies of the plasma fatty acids and colorectal cancer.
