# Supplementary material for: Comprehensive Investigation on Associations between Dietary Intake and Blood Levels of Fatty Acids and Colorectal Cancer Risk
Source: Nutrients. 2023 Feb 1;15(3):730. doi: 10.3390/nu15030730 (PMC9919095; doi:10.3390/nu15030730)
Supplement: Supplementary file 1 [file nutrients-15-00730-s001.zip › nutrients-2171127-supplementary FINAL 6.02.pdf]

nutrients

Comprehensive Investigation on Associations between Dietary Intake and Blood Levels of Fatty Acids and Colorectal Cancer Risk

Ying Lu [1,†] Doudou Li [2,†], Lijuan Wang [1,3], Han Zhang [2], Fangyuan Jiang [1], Rongqi Zhang [1], Liying Xu [1], Nan Yang [1], Shuhui Dai [1], Xiaolin Xu [1], Evropi Theodoratou [3,4,†] and Xue Li [1,5,*]

[1] The Key Laboratory of Intelligent Preventive Medicine of Zhejiang Province, Department of Big Data in Health Science, School of Public Health, Centre of Clinical Big Data and Analytics of The Second Affiliated Hospital, Zhejiang University School of Medicine, Hangzhou 310058, China
[2] College of Public Health, Zhengzhou University, Zhengzhou 450001, China
[3] Centre for Global Health, Usher Institute, University of Edinburgh, Edinburgh EH8 9AG, UK
[4] Cancer Research UK Edinburgh Centre, Medical Research Council Institute of Genetics and Cancer, University of Edinburgh, Edinburgh EH4 2XU, UK
[5] The Key Laboratory of Intelligent Preventive Medicine of Zhejiang Province, Hangzhou 310058, China
† These authors contributed equally to this work.
Correspondence: xue.li@ed.ac.uk

Supplementary Materials

Supporting information

Table S1. Search strategy.

Table S2. Results of quality assessment obtained with the Newcastle-Ottawa Scale for cohort studies.

Table S3. Results of quality assessment obtained with the Newcastle-Ottawa Scale for case control studies.

Table S4. Characteristics of the included studies in the meta-analysis of the intake of fatty acids and colorectal cancer.

Table S5. Characteristics of the included studies in the meta-analysis.

Table S6. Characteristics of the included Mendelian Randomization studies of the plasma fatty acids and colorectal cancer.

Supplementary Table S1. Search strategy.

**Medline**	
1	exp Colorectal Neoplasms/
2	exp Colonic Neoplasms/
3	exp Rectal Neoplasms/
4	((rectal or rectum or colonic or colon or colorectal or bowel* or sigmoid or intestin*) adj3 (cancer* or carcinoma* or neoplas* or tumor* or tumour* or adenocarcinoma* or adeno?carcinoma* or adenom* or lesion* or CRC)).mp.
5	1 or 2 or 3 or 4
6	exp Fatty Acids/
7	exp Fatty Acids, Omega-6/ or exp Fatty Acids, Omega-3/
8	((Aliphatic or palmitic or stearic or oleic or docosapentaenoic or eicosapentaenoic or docosahexanoic or alpha-Linolenic Acid or Arachidonic or Linoleic or palmitoleic) adj3 acid).mp.
9	(DPA or EPA or DHA or ALA or AA or MUFA or PUFA).mp.
10	6 or 7 or 8 or 9
11	5 and 10
**Embase**	
1	((Aliphatic or palmitic or stearic or oleic or docosapentaenoic or eicosapentaenoic or docosahexanoic or alpha-Linolenic Acid or Arachidonic or Linoleic or palmitoleic) adj3 acid).mp.
2	(DPA or EPA or DHA or ALA or AA or MUFA or PUFA or omega 3 or omega 6).mp.
3	exp colon carcinoma/ or exp colorectal cancer/ or exp rectum tumor/
4	((rectal or rectum or colonic or colon or colorectal or bowel* or sigmoid or intestin*) adj3 (cancer* or carcinoma* or neoplas* or tumor* or tumour* or adenocarcinoma* or adeno?carcinoma* or adenom* or lesion* or CRC)).mp.
5	3 or 4
6	fatty acid/
7	1 or 2 or 6
8	5 and 7

Supplementary Table S2. Results of quality assessment obtained with the Newcastle-Ottawa Scale for cohort studies.

Author name	Study design	Selection				Comparability	Outcome			Final score
		Representativeness of the exposed cohort	Selection of the non-exposed cohort	Ascertainment of exposure	Demonstration that outcome of interest was not present at start of study		Assessment of outcome	Was follow-up long enough for outcomes to occur	Adequacy of follow up of cohorts	
Willett (1990)	CO	0	1	1	1	2	1	1	1	8
Bostick (1994)	CO	0	1	1	1	1	1	1	1	7
Chyou (1996)	CO	0	1	1	1	1	1	1	1	7
Pietinen (1999)	CO	0	1	1	1	1	1	1	1	7
Järvinen (2001)	CO	1	1	1	1	1	1	1	1	8
Flood (2003)	CO	0	1	1	1	1	1	1	1	8
Lin (2004)	CO	0	1	1	1	1	1	1	1	7
Kobayashi (2004)	CO	1	1	1	1	1	1	1	1	8
Larsson (2005)	CO	0	1	1	1	2	1	1	1	8
Oba (2006)	CO	1	1	1	1	2	1	1	1	9
Weijenberg (2007)	CO	1	1	1	1	1	1	1	1	8
Hall (2008)	CO	0	1	1	1	2	1	1	1	8
Limburg (2008)	CO	0	1	1	1	1	1	1	1	7
Butler (2009)	CO	1	1	1	1	2	1	1	1	9
Daniel (2009)	CO	1	1	1	1	2	1	1	1	9
Lee (2009)	CO	0	1	1	1	1	1	1	1	7
Murff (2009)	CO	0	1	1	1	2	1	1	1	8
Sasazuki (2011)	CO	0	1	1	1	1	1	1	1	7
Kantor (2014)	CO	0	1	1	1	2	1	1	1	8
Song (2014)	CO	1	1	1	1	2	1	1	1	9
Kraja (2015)	CO	0	1	1	1	2	1	1	1	8
Navarro (2016)	CO	0	1	1	1	1	1	1	1	7
Shin (2020)	CO	0	1	1	1	2	1	1	1	8
Aglago (2020)	CO	1	1	1	1	2	1	1	1	9
Aglago (2021)	CO	1	1	1	1	2	1	1	1	9
Nguyen (2021)	CO	0	1	1	1	2	1	1	1	8
Wan (2022)	CO	0	1	1	1	2	1	1	1	8
Ayako (2022)	CO	0	1	1	1	2	1	1	1	8

[1]CO= cohort study

Supplementary Table S3. Results of quality assessment obtained with the Newcastle-Ottawa Scale for case control studies.

Author name	Study design	Selection				Comparability	Outcome			Final score
		Is the case definition adequate?	Representativeness of the cases	Selection of Controls	Definition of Controls		Ascertainment of exposure	Same method of ascertainment for cases and controls	Non-Response rate	
**Jain (1980)**	CC	1	1	1	1	1	1	1	0	7
**Zaridze (1992)**	CC	0	1	1	1	1	1	1	0	6
**Ghadirian (1997)**	CC	0	1	1	1	1	1	1	0	6
**Slattery (1997)**	CC	1	1	0	1	1	1	1	1	7
**Slattery (2001)**	CC	1	1	1	1	1	1	1	0	7
**Nkondjock (2003)**	CC	1	1	1	1	1	1	1	0	7
**Koh (2004)**	CC	1	1	0	1	1	1	1	0	6
**Brink (2004)**	CC	1	1	1	1	1	1	1	0	7
**Kojima (2005)**	CC	1	1	1	1	1	1	1	0	7
**Hall (2007)**	CC	0	1	0	1	1	1	1	0	5
**Kimura (2007)**	CC	1	1	1	1	1	1	1	0	7
**Theodoratou (2007)**	CC	1	1	1	1	2	1	1	0	8
**Vinikoor (2009)**	CC	1	1	0	1	1	1	1	0	6
**Vinikoor (2009)**	CC	1	1	0	1	1	1	1	0	6
**Dahm (2010)**	CC	0	1	1	1	2	1	1	0	7
**Kato (2010)**	CC	1	1	1	1	2	1	1	0	8
**Kim (2010)**	CC	1	1	1	1	1	1	1	0	7
**Hu (2011)**	CC	0	1	1	1	2	1	1	0	7
**Key (2012)**	CC	0	1	0	1	1	1	1	0	5
**Zhong (2013)**	CC	1	1	0	1	1	1	1	0	6
**Hodge (2015)**	CC	1	1	0	1	1	1	1	0	6
**Zhang (2015)**	CC	1	1	0	1	1	1	1	0	6
**Butler (2017)**	CC	1	1	0	1	1	1	1	0	6
**Mo (2018)**	CC	1	1	0	1	1	1	1	0	6
**Monireh (2022)**	CC	1	1	1	1	1	1	1	0	7

**Monireh (2022)**	CC	1	1	1	1	1	1	1	0	7

[1]CC=case-control study

Supplementary Table S4. Characteristics of the included studies in the meta-analysis of the intake of fatty acids and colorectal cancer.

Author (year) study type	Measurement(dietary)	Gender	Outcome types	Cases(n)	Participants(n)	Fatty acids	Adjusted RR or OR or HR (95% CI) (highest vs lowest intake)	Adjustment for confounding
Jain (1980) CC	Food frequency questionnaire (FFQ)	Men (Residents)	CRC	285	570	SFA	2.30(1.50-3.90)	
		Women (Residents)	CRC	257	514	SFA	3.50(2.20-6.40)	
		Man (Hospital)	CRC	285	547	SFA	1.70(1.30-2.40)	
		Women (Hospital)	CRC	257	530	SFA	2.00(1.40-2.70)	
Willett (1990) CO	61-item semiquantitative FFQ	Women	Colon cancer	150	88751	SFA<br>MUFA<br>LA	1.39(0.83-2.33)<br>1.72(1.01,2.93)<br>0.93(0.55,1.58)	Adjusted for total energy intake by regression analysis to 1600 kcal per day
Zaridze (1992) CC	132- item FFQ	Men & Women	CRC	217	434	TFA<br>SFA<br>MUFA<br>PUFA	0.42(0.13-1.34)<br>1.56(0.59-4.18)<br>0.54(0.20-1.51)<br>0.29(0.13-0.64)	Energy intake and education.
Bostick (1994) CO	127-item FFQ	Women	Colon cancer	212	35216	Total FA<br>SFA<br>MUFA<br>PUFA<br>N-3PUFA	0.86(0.54-1.33)<br>1.21(0.78-1.29)<br>0.85(0.54-1.35)<br>0.74(0.49-1.12)<br>0.70(0.45-1.09)	Covariates for final models for reported relative risks: age, total energy intake, height, parity, total vitamin E intake, a total vitamin E by age interaction term, and vitamin A supplement intake.

Chyou (1996) CO	24 hours diet recall	Men	Colon cancer	330	7945	PUFA	0.73(0.53-1.00)	Age-adjusted
			Rectal cancer	123	7945	PUFA	1.47(0.88-2.47)	
Ghadirian (1997) CC	Validated dietary questionnaire including >200 food items.	Men & Women	Colon cancer	402	1070	TFA<br>SFA<br>MUFA<br>PUFA	0.78(0.53-1.14)<br>0.71(0.49-1.03)<br>0.89(0.61-1.30)<br>0.96(0.65-1.42)	Gender, age, marital status, history of colon carcinoma in first-degree relatives, and total energy intake.
Pietinen (1999) CO	276-item diet questionnaire	Men	CRC	185	27111	TFA<br>SFA<br>MUFA<br>PUFA<br>Trans-FA<br>LA<br>N-3PUFA	0.90(0.60-1.30)<br>0.90(0.60-1.40)<br>1.20(0.80-1.80)<br>1.40(0.90-2.10)<br>1.10(0.70-1.60)<br>1.40(0.90-2.10)<br>1.20(0.80-1.90)	Adjusted for age (5-year categories) and supplement group, smoking years, body mass index, alcohol, education, physical activity at work, and calcium intake.
Järvinen (2001) CO	FFQ	Men & Women	CRC	109	9959	SFA<br>MUFA<br>PUFA	1.47(0.56-3.83)<br>2.37(0.86-2.51)<br>1.13(0.56-2.26)	Adjusted for age, sex, body mass index, occupation, smoking, geographical area, energy intake and consumption of vegetables, fruits and cereals.
			Colon cancer	63	9959	SFA<br>MUFA<br>PUFA	1.56(0.44-5.48)<br>2.37(0.61-9.19)<br>0.97(0.38-2.46)	
			Rectum cancer	46	9959	SFA<br>MUFA<br>PUFA	1.39(0.31-6.41)<br>2.38(0.52-10.85)	

							1.35(0.47-3.89)	
Slattery (2001) CC	The CARDIA diet history questionnaire	Men	Colon cancer	1099	2389	trans-FA<br>cis-FA	1.20(0.90-1.70)<br>1.00(0.80-1.30)	Age, body mass index, physical activity, energy, and dietary fiber and calcium intake. Models for women also are adjusted for estrogen status.
		Women	Colon cancer	894	2014	trans-FA<br>cis-FA	1.50(1.10-2.00)<br>1.00(0.80-1.40)	
Flood (2003) CO	62-item National Cancer Institute/Block FFQ	Women	CRC	487	45496	TFA<br>SFA<br>UFA	1.14(0.86-1.53)<br>0.87(0.60-1.27)<br>1.06(0.70-1.62)	Adjusting for energy using the multivariate nutrient density method and also controlling for total fat.
Kobayashi (2004) CO	FFQ	Men (AFRICAN AMERICANS)	Colon cancer	300	57591	EPA<br>DHA<br>Ln-3/n-6 PUFA	1.06(0.71-1.58)<br>0.98(0.66-1.46)<br>1.08(0.74-1.56)	Age, area, family history of colorectal cancer, BMI, physical activity, smoking status, alcohol intake, use of vitamin supplement, total energy, cereal, vegetable, and meat intake.
			Rectum cancer	154	57591	EPA<br>DHA<br>Ln-3/n-6 PUFA	1.37(0.81-2.32)<br>1.17(0.70-1.96)<br>1.26(0.77-2.05)	
		Women (AFRICAN AMERICANS)	Colon cancer	156	59103	EPA<br>DHA<br>Ln-3/n-6 PUFA	1.04(0.60-1.80)<br>1.08(0.63-1.87)<br>0.92(0.55-1.54)	
			Rectum cancer	95	59103	EPA<br>DHA<br>Ln-3/n-6 PUFA	0.57(0.29-1.15)<br>0.66(0.33-1.33)<br>0.61(0.31-1.19)	
Brink (2004) CO	150-item semi-quantitative FFQ	Men & Women	Colon cancer	448	3346	TFA<br>SFA<br>MUFA<br>PUFA	0.95(0.71-1.27)<br>0.93(0.69-1.26)<br>0.98(0.73-1.33)<br>1.21(0.89-1.64)	Relative risk adjusted for age, sex, QI, smoking, energy intake and family history of CRC

						ALA	1.01(0.75-1.36)	
						LA	1.31(0.97-1.77)	
			Rectum cancer	160	3346	TFA	0.86(0.55-1.36)	
						SFA	0.73(0.46-1.17)	
						MUFA	0.87(0.56-1.37)	
						PUFA	0.83(0.53-1.29)	
						ALA	0.91(0.58-1.44)	
						LA	1.03(0.66-1.62)	
Koh (2004) CC	24-h food recall	Men & Women	CRC	310	1487	n-6 PUFA	1.04(0.63-1.70)	Age at recruitment, year of recruitment, gender, dialect group, level of education, body mass index, smoking status, frequency of alcohol consumption, family history of colorectal cancer and total energy intake.
			Colon cancer	180	1357	n-6 PUFA	1.04(0.56-1.92)	
			Rectum cancer	130	1307	n-6 PUFA	0.99(0.49-1.99)	
Larsson (2005) CO	67-item FFQ.	Women	CRC	798	60708	Linoleic acid	0.71(0.55-0.91)	Age (in mo), body mass index (in kg/m2; <23, 23–24.9, 25–29.9, and ≥30), education (less than high school, high school graduate, or more than high school), total energy intake (continuous), and quintiles of intakes of folate, vitamin B-6, cereal fiber, and red meat.
			Proximal Colon cancer	246	60708	Linoleic acid	0.82(0.53-1.28)	
			Distal Colon cancer	170	60708	Linoleic acid	0.53(0.31-0.92)	
			Rectum cancer	249	60708	Linoleic acid	0.75(0.49-1.13)	
Oba (2006) CO	169-item semi-quantitative FFQ	Men	Colon cancer	111	13894	TFA	1.36(0.83-2.24)	RR adjusted for age, height, BMI, total pack-years of cigarette smoking, alcohol intake, and physical activity.
						SFA	1.04(0.65-1.66)	
						MUFA	1.25(0.78-1.99)	

						PUFA	1.65(1.00-2.74)	
						LCn-3PUFA	1.24(0.80-1.95)	
		Women		102	16327	TFA	0.77(0.47-1.27)	
						SFA	0.85(0.53-1.36)	
						MUFA	0.87(0.53-1.44)	
						PUFA	0.72(0.44-1.18)	
						LCn-3PUFA	0.89(0.56-1.44)	
Kimura (2007) CC	148-item dietary questionnaires	Men & Women	CRC	782	1575	TFA	0.77(0.53-1.13)	Age, sex, residential area, body mass index 10 years before, parental colorectal cancer, smoking, alcohol use, type of job, leisure-time physical activity, dietary calcium and dietary fiber.
						SFA	1.04(0.71-1.51)	
						MUFA	0.88(0.62-1.25)	
						n-6 PUFA	0.77(0.54-1.10)	
						n-3 PUFA	0.74(0.52-1.06)	
			Proximal Colon cancer	177	970	TFA	0.90(0.48-1.70)	
						SFA	0.99(0.52-1.88)	
						MUFA	0.99(0.56-1.73)	
						n-6 PUFA	1.29(0.70-2.35)	
						n-3 PUFA	0.84(0.45-1.55)	
			Distal Colon cancer	262	1055	TFA	0.70(0.41-1.20)	
						SFA	1.17(0.69-1.99)	
						MUFA	0.92(0.55-1.55)	
						n-6 PUFA	0.67(0.40-1.12)	
						n-3 PUFA	0.56(0.34-0.92)	
			Rectum cancer	327	1120	TFA	0.78(0.48-1.27)	
						SFA	1.00(0.62-1.64)	
						MUFA	0.84(0.53-1.34)	
						n-6 PUFA	0.69(0.43-1.10)	

						n-3 PUFA	0.88(0.56-1.41)	
Hall (2008) CO	Abbreviated semiquantitative FFQ	Men	CRC	500	21409	n-3 PUFA	0.76(0.59-0.98)	Age, smoking (never smoked, past smoking, current smoking), body-mass index (<23, 23 to 24.99, 25 to 26.99, ≥ 27), multivitamin use (never use, past use, current use), history of diabetes, random assignment to aspirin or placebo, vigorous exercise (< once per week, 1–4 times per week, ≥ 5–6 times per week), alcohol intake (≤ once per week, 2–6 times per week, ≥ once per day), and quartile of red meat intake.
Limburg (2008) CO	126-item semi-quantitative FFQ	Women	CRC	1229	35216	trans-FA	1.06(0.88-1.28)	Age, total energy intake, body mass index, physical activity level, estrogen use, self-reported diabetes mellitus, smoking status, and intake of total fat, red meat, fruits and vegetables, calcium, folate, vitamin E and alcohol.
			Proximal CRC	631	35216	trans-FA	1.04(0.80-1.35)	
			Distal CRC	571	35216	trans-FA	1.09(0.84-1.43)	
Lee (2009) CO	A validated, quantitative FFQ	Women	CRC	394	73224	TFA	1.10(0.70-1.70)	Adjusted for age, education, income, survey season, tea consumption, NSAID use, energy intake, and fiber intake.
						SFA	1.10(0.70-1.60)	
						MUFA	0.90(0.60-1.30)	
						PUFA	0.80(0.50-1.30)	
			Colon cancer	236	73224	TFA	1.40(0.80-2.30)	
						SFA	1.20(0.80-2.10)	
						MUFA	1.00(0.60-1.70)	
						PUFA	1.20(0.70-2.10)	
			Rectum	158	73224	TFA	0.80(0.40-1.50)	

			cancer			SFA<br>MUFA<br>PUFA	0.80(0.40-1.50)<br>0.70(0.40-1.30)<br>0.50(0.30-0.90)	
Vinikoor (2009) CC	100-item semi-quantitative Block FFQ	Men & Women (WHITES)	Colon cancer	334	920	trans-FA	1.01(0.69-1.49)	Age, sex, energy intake, and calcium intake.
			Proximal Colon cancer	140	726	trans-FA	1.05(0.60-1.83)	Age, sex, energy intake, calcium intake, and alcohol intake.
			Distal Colon cancer	159	745	trans-FA	0.92(0.55-1.56)	
		Men & Women (AFRICAN AMERICANS)	Colon cancer	276	679	trans-FA	0.99(0.61-1.62)	Age, sex, energy intake, and calcium intake.
			Proximal Colon cancer	123	526	trans-FA	1.35(0.71-2.55)	Age, sex, energy intake, calcium intake, and alcohol intake.
			Distal colon cancer	123	526	trans-FA	0.69(0.36-1.31)	
Vinikoor (2009) CC	100-item semi-quantitative Block FFQ	Men & Women WHITES	Distal colon cancer	717	1516	trans-FA	1.45(1.04-2.03)	Adjusted for energy intake, energy-adjusted total fatty acid consumption, age, sex, BMI 1 year prior, highest level of education attained, and family history of colorectal cancer
			Rectum cancer	265	1064	trans-FA	1.30(0.81,2.08)	

			Sigmoid	314	1113	trans-FA	1.29(0.83,2.02)	
			Rectosigmoid	128	927	trans-FA	1.51(0.81,2.81)	
		Men & Women (AFRICAN AMERICANS)	Distal colon cancer	233	392	trans-FA	0.87(0.42,1.81)	
			Rectum cancer	81	240	trans-FA	0.88(0.33,2.85)	
			Sigmoid	122	281	trans-FA	0.90(0.37,2.17)	
			Rectosigmoid	28	187	trans-FA	0.93(0.22,3.89)	
Dahm (2010) CC	4- to 7-day food diaries and FFQ	Men & Women food dairy	CRC	576	2572	TFA<br>SFA<br>MUFA<br>PUFA	0.83(0.57-1.20)<br>0.73(0.49-1.07)<br>0.71(0.49-1.04)<br>0.98(0.68-1.43)	Adjusted for age, height, weight, non-fat energy (MJ) intake, fiber, alcohol, physical activity level, social class, educational level and smoking status.
		FFQ	CRC	496	2305	TFA<br>SFA<br>MUFA<br>PUFA	0.74(0.50-1.09)<br>0.82(0.55-1.21)<br>0.91(0.62-1.34)<br>0.95(0.66-1.37)	
Kato (2010)	A validated semi-	Men & Women	CRC	1163	2664	trans-FA	0.84(0.67-1.06)<br>0.95(0.75-1.21)	Age, gender, specimen type (blood vs. others), total calcium and dietary fiber intake, physical

CC	quantitative FFQ						0.94(0.74-1.18)<br>0.81(0.65-1.02)<br>0.92(0.73-1.15)<br>1.17(0.93-1.48)	activities in their 30s, body mass index, family history of colorectal cancer, pack-years of cigarette smoking and postmenopausal hormone use (women), highest education achieved and NSAID use.
Hu (2011) CC	69-item semi-quantitative FFQ	Men & Women	Colon cancer	1727	6766	trans-FA	1.38(1.08-1.78)	Age group (20–49, 50–59, 60–69, 70–76 years), province, education, body mass index (< 25, 25–29.9, Z 30 kg/m2), sex, alcohol drinking (g/day), packyear smoking, total of vegetable and fruit intake (servings/week), monounsaturated fat, polyunsaturated fat and total energy intake
			Rectum cancer	1447	6486	trans-FA	1.06(0.81-1.37)	
Key (2012) CC	Seven-day food diaries	Men & Women	CRC	547	2415	n-6 PUFA<br>n-3 PUFA<br>n-6/n-3PUFA	1.09(0.77-1.55)<br>0.82(0.59-1.13)<br>1.22(0.92-1.63)	Age, date of diary and sex, and adjusted for exact age, height, weight, energy intake, alcohol intake, fiber intake, smoking, education, social class, physical activity.
Kantor (2014) CO	120-item FFQ	Men & Women	CRC	488	68109	EPA<br>DHA<br>EPA+DHA	0.99(0.74-1.33)<br>0.89(0.66-1.21)<br>0.92(0.68-1.24)	Age, sex, race/ethnicity, education, BMI, energy intake, MET-hours per week of moderate/vigorous activity, alcohol intake, smoking history, multivitamin use, calcium intake, dietary fiber intake, fruit and vegetable intake, red/processed meat intake, aspirin use, non-aspirin NSAID use, family history of colorectal cancer, history of sigmoidoscopy/colonoscopy, history of polyps, hormone replacement therapy,

								cardiovascular disease, memory loss, use of cholesterol-lowering drugs, and omega-6 (linoleic +arachidonic) intake
Kraja (2015) CO	170-item semi-quantitative FFQ	Men & Women	CRC	222	4967	Total PUFA<br>n-6 PUFA<br>n-3 PUFA<br>SFA	0.95(0.60-1.32)<br>0.89(0.65-1.23)<br>1.44(1.02-2.04)<br>1.13(0.79-1.62)	Age, gender, energy-adjusted DF intake, and Dutch Healthy Diet index (excluding PUFA, fish, SFA, and DF components).
Navarro (2016) CO	122-item FFQ	Women	CRC	1952	134017	TFA<br>N-6PUFA<br>N-3PUFA	0.98(0.76-1.27)<br>0.84(0.68-1.05)<br>0.90(0.74-1.09)	The following variables were included in both the multivariate and continuous models: total energy intake (continuous), age (continuous), body mass index (continuous), education (high school, technical school or some college, college graduate or post-graduate), family history of colorectal cancer (yes/no), history of colonoscopy (yes/no), current NSAID use (yes/no), alcohol intake (continuous), smoking history (never, former, current), physical activity (total metabolic equivalent-hours, continuous), ever use of hormone therapy (never, current/former), folate (DFE µg/day, continuous), calcium (mg/day, continuous), and red meat intake (g/day, continuous), and study component (OS, CT) and CT randomization assignment and treatment arm.
Mo 2018	70-item BFFQ	Men & Women	Proximal Colon	72	108	n-6 PUFA<br>n-3 PUFA	2.58(1.22-5.45)<br>2.10(1.11-3.97)	Age, gender, physical activity, and distal ACF number.

CC			cancer			n-6/ n-3 PUFA	0.79(0.44-1.44)	
Slattery (1997) CC	Validated CARDIA diet history questionnaire	Men	Colon cancer	1099	2389	TFA	0.79(0.58-1.09)	Age at diagnosis or selection, total energy intake, dietary fiber, cholesterol, calcium, BMI, physical activity, family history of colorectal cancer and use of NSAIDs.
						SFA	0.88(0.64-1.22)	
						PA	0.77(0.55-1.09)	
						MUFA	0.89(0.65-1.21)	
						OA	0.94(0.70-1.27)	
						PUFA	1.07(0.82-1.41)	
						LA	1.12(0.85-1.47)	
						AA	1.17(0.85-1.61)	
						EPA	1.13(0.87-1.49)	
						n-3PUFA	1.00(0.76-1.31)	
						n-3/ n-6PUFA	0.99(0.76-1.29)	
		Women	Colon cancer	894	2014	TFA	1.01(0.71-1.43)	
						SFA	0.96(0.67-1.37)	
						PA	0.92(0.63-1.35)	
						MUFA	0.94(0.66-1.34)	
						OA	0.91(0.64-1.28)	
						PUFA	1.05(0.77-1.43)	
						LA	1.07(0.79-1.46)	
						AA	0.98(0.70-1.37)	
						EPA	0.90(0.67-1.21)	
						n-3	0.89(0.66-1.22)	
						n-3/ n-6PUFA	0.95(0.71-1.28)	
Nkondjock (2003) CC	200-item FFQ	Men	CRC	200	439	Butyric acid	0.87(0.51-1.48)	Age, marital status, history of colorectal cancer in first-degree relatives, body mass index one year prior to diagnosis, and physical
						Caprylic acid	0.88(0.40-1.10)	
						Capric acid	0.96(0.56-1.65)	

				Myristic acid	1.01(0.59-1.72)	activity.
				PA	1.09(0.62-1.03)	
				SA	1.26(0.73-2.19)	
				POA	1.10(0.62-1.96)	
				OA	0.76(0.44-1.32)	
				LA	1.17(0.68-2.01)	
				ALA	0.94(0.55-1.60)	
				AA	2.03(1.16-3.54)	
				EPA	0.81(0.48-1.38)	
				DPA	0.82(0.48-1.41)	
				DHA	1.01(0.60-1.69)	
				n-6 PUFA	1.22(0.71-2.10)	
				n-3 PUFA	0.99(0.58-1.67)	
				n-6/n-3PUFA	1.22(0.71-2.10)	
				trans-FA	0.88(0.50-1.56)	
				SFA	1.19(0.68-2.10)	
				MUFA	0.88(0.51-1.52)	
				PUFA	1.09(0.66-1.79)	
Women	CRC	202	631	Butyric acid	0.54(0.34-0.96)	
				Caprylic acid	0.66(0.40-1.10)	
				Capric acid	0.70(0.42-1.16)	
				Myristic acid	0.71(0.43-1.17)	
				PA	0.95(0.57-1.56)	
				SA	1.15(0.70-1.88)	
				POA	1.02(0.62-1.71)	
				OA	1.65(0.99-2.75)	

				LA	1.09(0.67-1.80)
				ALA	0.78(0.46-1.32)
				AA	1.89(1.11-3.22)
				EPA	1.56(0.94-2.58)
				DPA	1.07(0.64-1.80)
				DHA	1.98(1.18-3.32)
				n-6 PUFA	1.15(0.70-1.89)
				n-3 PUFA	0.84(0.50-1.41)
				n-6/n-3PUFA	1.47(0.86-2.50)
				trans-FA	1.00(0.61-1.65)
				SFA	0.87(0.53-1.41)
				MUFA	1.48(0.89-2.48)
				PUFA	1.24(0.75-2.04)
Men & Women	CRC	402	1070	Butyric acid	0.74(0.51-1.05)
				Caprylic acid	0.74(0.52-1.05)
				Capric acid	0.82(0.57-1.16)
				Myristic acid	0.84(0.59-1.20)
				PA	1.03(0.72-1.48)
				SA	1.04(0.72-1.48)
				POA	1.03(0.71-1.47)
				OA	1.04(0.73-1.48)
				LA	1.08(0.76-1.53)
				ALA	0.71(0.49-1.02)
				AA	2.11(1.47-3.06)
				EPA	1.06(0.76-1.52)
				DPA	0.88(0.62-1.26)

						DHA	1.34(0.94-1.91)	
						n-6 PUFA	1.07(0.76-1.54)	
						n-3 PUFA	0.73(0.51-1.05)	
						n-6/n-3PUFA	1.61(1.11-2.34)	
						trans-FA	0.83(0.58-1.19)	
						SFA	0.97(0.68-1.38)	
						MUFA	0.99(0.69-1.41)	
						PUFA	1.04(0.74-1.48)	
Lin (2004) CO	131-item FFQ	Women	CRC	202	37547	TFA	1.00(0.63-1.58)	Age, random treatment assignment, body mass index, family history of colorectal cancer, history of colorectal polyps, physical activity, cigarette smoking, alcohol consumption, postmenopausal hormone therapy, and total energy intake in a multivariate model.
						SFA	0.92(0.61-1.41)	
						MUFA	1.09(0.68-1.73)	
						n-6PUFA	1.60(0.98-2.60)	
						n-3PUFA	1.11(0.73-1.69)	
						Trans unsaturated	1.30(0.89-2.05)	
						Butyric acid	1.19(0.76-1.86)	
						PA	0.99(0.63-1.55)	
						SA	0.87(0.56-1.35)	
						POA	0.67(0.42-1.08)	
						OA	1.16(0.74-1.84)	
						LA	1.42(0.87-2.32)	
						AA	0.90(0.59-1.36)	
Theodoratou (2007) CC	150-item semi-quantitative FFQ	Men & Women	CRC	1458	2899	TFA	0.91(0.70-1.18)	Family history of colorectal cancer, total energy intake (residual method), total fiber intake, alcohol intake, use of nonsteroidal antiinflammatory drugs, smoking, body mass index, physical activity, total energy intake and
						SFA	1.07(0.73-1.56)	
						MUFA	1.18(0.76-1.82)	
						PUFA	0.82(0.63-1.07)	
						n-6PUFA	0.92(0.71-1.18)	

						n-3PUFA	0.56(0.44-0.72)	intake of total fatty acids.
						trans-FA	1.15(0.85-1.55)	
						n-3/n-6PUFA	0.63(0.49-0.80)	
						PA	1.21(0.81-1.81)	
						SA	1.71(1.18-2.49)	
						OA	1.48(1.00-2.19)	
						LA	0.91(0.71-1.17)	
						AA	0.93(0.72-1.19)	
						ALA	0.80(0.62-1.03)	
						EPA	0.57(0.45-0.73)	
						DHA	0.60(0.47-0.76)	
Weijenberg (2007) CO	150–item semi-quantitative FFQ	Men & Women	Colon cancer	434	3382	TFA	0.96(0.72-1.28)	Age, sex, body mass index, smoking, energy intake and family history of colorectal cancer.
						SFA	0.94(0.69-1.27)	
						MUFA	0.99(0.73-1.34)	
						PUFA	1.21(0.89-1.63)	
						LA	1.30(0.96-1.77)	
			Rectum cancer	154	3102	TFA	0.87(0.55-1.36)	
						SFA	0.74(0.46-1.18)	
						MUFA	0.88(0.56-1.37)	
						PUFA	0.82(0.53-1.29)	
						LA	1.03(0.65-1.62)	
Butle (2009) CO	165-item quantitative FFQ	Men	CRC(Localized)	210	27293	TFA	0.90(0.59-1.38)	Age at interview (yr), dialect group (Cantonese, Hokkien), interview year (1993–1995, 1996–1998), diabetes at baseline (no, yes), smoking history (never, "heavy" or 13 cigarettes per day starting age <15 years,
						SFA	0.85(0.56-1.30)	
						MUFA	1.07(0.72-1.60)	
						Total PUFA	0.97(0.65-1.44)	
						n-3 PUFA	0.78(0.51-1.21)	

					Marine n-3 PUFA	1.40(0.94-2.08)	“light” or non heavy smokers), body mass index (<20, 20–23.9, 24–27.9, 28 m/kg2), alcohol intake (0, <7, 7 drinks/week), education (no formal education, primary school, secondary school or higher), any weekly physical activity (no, yes), first degree relative diagnosed with colorectal cancer (no, yes), and total daily energy intake (kcal). CI 5 confidence interval.
					n-6 PUFA	0.92(0.62-1.37)	
					n-3/n-6PUFA	0.93(0.62-1.41)	
					Marine n-3/n-6	1.08(0.73-1.59)	
		CRC(Advanced)	291	27293	TFA	0.70(0.49-1.00)	
					SFA	0.76(0.54-1.07)	
					MUFA	0.78(0.55-1.11)	
					Total PUFA	0.86(0.61-1.20)	
					n-3 PUFA	1.09(0.80-1.50)	
					Marine n-3 PUFA	1.27(0.93-1.75)	
					n-6 PUFA	0.85(0.61-1.19)	
					n-3/n-6PUFA	1.32(0.94-1.84)	
					Marine n-3/n-6	1.54(1.09-2.16)	
	Women	CRC(Localized)	166	34028	TFA	1.86(1.18-2.92)	
					SFA	1.69(1.08-2.63)	
					MUFA	1.72(1.09-2.70)	
					Total PUFA	0.91(0.58-1.43)	
					n-3 PUFA	1.19(0.75-1.89)	
					Marine n-3 PUFA	0.70(0.45-1.07)	
					n-6 PUFA	0.91(0.58-1.42)	
					n-3/n-6PUFA	0.82(0.53-1.28)	
					Marine n-3/n-6	0.86(0.56-1.32)	
		CRC(Advanced)	236	34028	TFA	0.88(0.60-1.30)	
					SFA	0.88(0.61-1.28)	
					MUFA	1.07(0.74-1.54)	
					Total PUFA	1.03(0.70-1.51)	

						n-3 PUFA	1.09(0.75-1.59)	
						Marine n-3 PUFA	1.46(1.00-2.12)	
						n-6 PUFA	1.01(0.69-1.47)	
						n-3/n-6PUFA	1.15(0.80-1.65)	
						Marine n-3/n-6	1.32(0.90-1.95)	
		Men & Women	CRC(Localized)	376	61321	Total PUFA	0.94(0.70-1.26)	Age at interview (yr), sex, dialect group (Cantonese, Hokkien), interview year (1993–1995, 1996–1998), diabetes at baseline (no, yes), smoking history(never, “heavy” or 13 cigarettes per day starting age <15 years, “light” or nonheavy smokers), body mass index (<20, 20–23.9, 24–27.9, 28 m/kg2), alcohol intake (0, <7, 7 drinks/week), education (no formal education, primary school, secondary school or higher), any weeklyphysical activity (no, yes), first degree relative diagnosed with colorectal cancer (no, yes), and total daily energy intake (kcal). CI 5 confidence interval.
						n-3 PUFA	0.97(0.71-1.32)	
						Marine n-3 PUFA	1.02(0.76-1.37)	
						n-6 PUFA	0.92(0.68-1.23)	
						n-3/n-6PUFA	0.88(0.65-1.19)	
						Marine n-3/n-6	0.98(0.73-1.30)	
			CRC(Advanced)	527	61321	Total PUFA	0.91(0.71-1.17)	
						n-3 PUFA	1.07(0.84-1.36)	
						Marine n-3 PUFA	1.33(1.05-1.70)	
						n-6 PUFA	0.90(0.70-1.16)	
						n-3/n-6PUFA	1.24(0.97-1.59)	
						Marine n-3/n-6	1.45(1.12-1.87)	
Daniel (2009) CO	152-item FFQ	Men	CRC	452	43108	n-6 PUFA	0.81(0.61-1.07)	Age, energy, HRT (in women only), recreational physical activity, NSAID use, colorectal screening, BMI, and red and processed meat, low-fat dairy, fruit, and vegetable intake.
						n-3 PUFA	0.86(0.66-1.13)	
						ALA	0.87(0.66-1.14)	
						Marine n-3	1.00(0.75-1.33)	
						n-6/n-3 PUFA	1.04(0.78-1.38)	
		Women	CRC	417	55972	n-6 PUFA	1.17(0.88-1.55)	
						n-3 PUFA	1.38(1.02-1.85)	

						ALA	1.38(1.02-1.85)	
						Marine n-3	0.94(0.72-1.24)	
						n-6/n-3PUFA	0.96(0.72-1.30)	
Murff (2009) CO	FFQ	Women	CRC	332	73243	LA	1.20(0.62-2.34)	Age, energy intake (kcal), total energy-adjusted n-3 PUFA intake (g/d), energy-adjusted ratio of total n-6 PUFA to n-3 PUFA intake, BMI (kg/m2), current smoker, alcohol use, regular physical activity in past 5 y, total energy-adjusted red meat intake (g/d), menopausal status, hormone replacement therapy use, multivitamin use, and aspirin use.
						AA	0.96(0.62-1.50)	
						Total n-6 PUFA	1.10(0.57-2.12)	
						ALA	1.03(0.52-2.05)	
						Total n-3 PUFA	1.16(0.56-2.39)	
						n-6/n-3PUFA	1.88(0.93-3.80)	
			Colon cancer	200	73243	LA	1.03(0.53-2.00)	
						AA	1.14(0.67-1.94)	
						Total n-6 PUFA	0.88(0.40-1.92)	
						ALA	1.40(0.58-3.37)	
						Total n-3 PUFA	1.51(0.61-3.74)	
						n-6/n-3PUFA	2.02(0.85-4.80)	
			Rectum cancer	132	73243	LA	2.05(0.61-6.86)	
						AA	0.68(0.31-1.52)	
						Total n-6 PUFA	1.37(0.55-3.41)	
						ALA	0.64(0.22-1.89)	
						Total n-3 PUFA	0.75(0.23-2.41)	
						n-6/n-3PUFA	1.62(0.48-5.50)	
Kim (2010) CC	124-item validated FFQ	Men & Women	Distal Large Bowel Cancer	929	1872	Total PUFA	0.99(0.59-1.67)	Age (40–44, 45–49, 50–54, 55–59, 60–64, 65–69, 70–74, 75–79 years), race, sex, daily energy intake, offset term, years of education (<12, 12–15, 16 years), endoscopic screening in the past 10 years (yes or no), red meat intake
						Total n-3 PUFA	0.87(0.52-1.47)	
						AA	0.83(0.53-1.31)	
						Total n-3 PUFA	0.96(0.61-1.51)	
						ALA	1.03(0.66-1.60)	

						EPA<br>DHA<br>n-6/n-3PUFA	0.65(0.47-0.91)<br>0.58(0.41-0.81)<br>1.08(0.78-1.49)	(<34.7, 34.7–<57.1, 57.1–<95.8, 95.8 g/day), vitamin E intake (<6.6, 6.6–<9.2, 9.2–<12.4, 12.4 mg/day), and body mass index 1 year prior (<25, 25–29.9, 30 kg/m2).
Sasazuki (2011) CO	138-item validated FFQ	Men	Colon cancer	521	41382	EPA<br>DHA<br>DPA<br>Marine n-3 PUFA<br>ALA<br>Total n-3<br>Total n-6<br>n-3/n-6PUFA	0.81(0.50-1.30)<br>1.02(0.61-1.70)<br>0.86(0.53-1.38)<br>0.96(0.57-1.61)<br>0.84(0.56-1.28)<br>0.76(0.48-1.18)<br>0.85(0.57-1.25)<br>1.22(0.81-1.85)	Age, area, BMI, smoking status, alcohol drinking, past history of or medication use of DM, METs, screening for CRC, total calorie, intake of calcium, vitamin D, fiber and red meat.
			Proximal Colon cancer	142	41382	EPA<br>DHA<br>DPA<br>Marine n-3 PUFA<br>ALA<br>Total n-3<br>Total n-6<br>n-3/n-6PUFA	0.27(0.11-0.66)<br>0.48(0.18-1.29)<br>0.35(0.14-0.88)<br>0.35(0.14-0.88)<br>0.61(0.27-1.34)<br>0.42(0.18-0.98)<br>0.46(0.21-0.99)<br>0.92(0.42-2.01)	
			Distal Colon cancer	197	41382	EPA<br>DHA<br>DPA<br>Marine n-3 PUFA<br>ALA<br>Total n-3	1.34(0.68-2.67)<br>1.83(0.80-4.17)<br>1.80(0.83-3.91)<br>1.82(0.79-4.20)<br>1.31(0.65-2.63)<br>1.07(0.52-2.20)	

				Total n-6	1.59(0.85-2.99)
				n-3/n-6 PUFA	1.23(0.65-2.34)
	Rectum cancer	253	41382	EPA	1.06(0.53-2.11)
				DHA	1.12(0.53-2.36)
				DPA	1.34(0.67-2.68)
				Marine n-3 PUFA	1.07(0.51-226)
				ALA	1.10(0.61-1.98)
				Total n-3	1.33(0.70-2.51)
				Total n-6	0.86(0.50-1.50)
				n-3/n-6 PUFA	1.62(0.89-2.93)
Women	Colon cancer	350	47192	EPA	0.49(0.27-0.89)
				DHA	0.50(0.28-0.90)
				DPA	0.53(0.29-1.00)
				Marine n-3 PUFA	0.60(0.31-1.14)
				ALA	1.01(0.65-1.57)
				Total n-3	0.68(0.41-1.12)
				Total n-6	0.87(0.57-1.31)
				n-3/n-6 PUFA	1.05(0.61-1.79)
	Proximal Colon cancer	171	47192	EPA	0.45(0.20-1.05)
				DHA	0.47(0.20-1.14)
				DPA	0.37(0.16-0.85)
				Marine n-3 PUFA	0.59(0.24-1.45)
				ALA	0.84(0.45-1.59)
				Total n-3	0.55(0.27-1.11)
				Total n-6	0.74(0.40-1.35)
				n-3/n-6 PUFA	1.07(0.50-2.30)

			Distal Colon cancer	88	47192	EPA	0.57(0.17-1.91)	
						DHA	0.62(0.17-2.22)	
						DPA	0.74(0.23-2.42)	
						Marine n-3 PUFA	0.61(0.17-2.24)	
						ALA	0.92(0.38-2.22)	
						Total n-3	0.81(0.28-2.33)	
						Total n-6	0.70(0.32-1.57)	
						n-3/n-6PUFA	1.20(0.40-3.62)	
			Rectum cancer	144	47192	EPA	0.74(0.30-1.82)	
						DHA	1.14(0.46-2.81)	
						DPA	1.49(0.57-3.87)	
						Marine n-3 PUFA	1.62(0.61-4.32)	
						ALA	1.02(0.50-2.06)	
						Total n-3	1.13(0.51-2.49)	
						Total n-6	0.81(0.42-1.56)	
						n-3/n-6 PUFA	1.00(0.44-2.27)	
Zhong (2013) CC	81-item FFQ	Men	CRC	278	834	TFA	0.95(0.61-1.48)	Adjusted for marital status, education, regular smoking, passive smoking, regular drinking, leisure-time physical activity, first-degree relative with cancer, total energy, total vegetable, and total fruit intakes.
						SFA	1.15(0.73-1.81)	
						MUFA	1.14(0.73-1.79)	
						Total n-3	0.35(0.21-0.56)	
						Total n-6	0.67(0.42-1.07)	
		Women	CRC	211	631	TFA	1.07(0.64-1.77)	Adjusted for marital status, education, regular smoking, passive smoking, regular drinking, leisure-time physical activity, first-degree relative with cancer, total energy, total vegetable, total fruit intakes and menopausal
						SFA	1.58(0.93-2.68)	
						MUFA	1.60(0.95-2.71)	
						Total n-3	0.59(0.35-0.98)	
						Total n-6	0.96(0.58-1.59)	

								status in women.
		Men & Women	CRC	489	1465	TFA	0.95(0.68-1.32)	Adjusted for marital status, education, regular
						SFA	1.25(0.89-1.75)	smoking, passive smoking, regular drinking,
						MUFA	1.29(0.93-1.80)	leisure-time physical activity, first-degree
						Total n-3	0.45(0.32-0.64)	relative with cancer, total energy, total
						Total n-6	0.79(0.56-1.10)	vegetable, and total fruit intakes.
						LA	0.78(0.55-1.09)	
						AA	1.23(0.88-1.72)	
						ALA	0.54(0.38-0.76)	
						EPA	0.58(0.41-0.82)	
						DPA	0.65(0.47-0.91)	
						DHA	0.69(0.50-0.97)	
			Colon cancer	267	1243	TFA	1.03(0.69-1.52)	
						SFA	1.27(0.84-1.92)	
						MUFA	1.44(0.96-2.16)	
						Total n-3	0.39(0.25-0.61)	
						Total n-6	0.76(0.50-1.14)	
			Rectum cancer	218	1194	TFA	0.83(0.53-1.30)	
						SFA	1.28(0.80-2.04)	
						MUFA	1.20(0.76-1.89)	
						Total n-3	0.56(0.36-0.88)	
						Total n-6	0.82(0.53-1.29)	
Song (2014) CO	validated 116- to 131-item FFQ	Men	CRC	987	47143	ALA	0.89(0.70-1.13)	Age, calendar year, family history of colorectal
						Marine n-3	1.05(0.85-1.30)	cancer, prior lower gastrointestinal endoscopy,
						Total n-6	1.17(0.95-1.44)	pack-years of smoking before age 30, BMI,
			Proximal	342	47143	ALA	0.81(0.53-1.23)	physical activity, current multivitamin use,

			Colon cancer			Marine n-3	1.03(0.72-1.47)	regular aspirin or NSAID use (2 tablets/week), total caloric intake, red meat, process meat, alcohol consumption and energy-adjusted intake of folate, calcium, vitamin D and total fiber.
						Total n-6	1.25(0.88-1.79)	
			Distal Colon cancer	301	47143	ALA	1.15(0.75-1.75)	
						Marine n-3	1.43(0.97-2.11)	
						Total n-6	0.97(0.66-1.41)	
			Rectum cancer	215	47143	ALA	0.68(0.41-1.15)	
						Marine n-3	0.79(0.51-1.22)	
						Total n-6	1.30(0.84-2.03)	
		Women	CRC	1469	76386	ALA	1.05(0.86-1.29)	Age, calendar year, family history of colorectal cancer, prior lower gastrointestinal endoscopy, pack-years of smoking before age 30, BMI, physical activity, current multivitamin use, postmenopausal status and hormone use, regular aspirin or NSAID use (2 tablets/week), total caloric intake, red meat, process meat, alcohol consumption and energy-adjusted intake of folate, calcium, vitamin D and total fiber.
						Marine n-3	1.03(0.89-1.20)	
						Total n-6	0.89(0.70-1.12)	
			Proximal Colon cancer	713	76386	ALA	1.04(0.78-1.40)	
						Marine n-3	0.86(0.69-1.08)	
						Total n-6	1.06(0.76-1.48)	
			Distal Colon cancer	416	76386	ALA	1.18(0.81-1.71)	
						Marine n-3	1.36(1.03-1.80)	
						Total n-6	0.74(0.48-1.15)	
			Rectum cancer	310	76386	ALA	0.84(0.52-1.37)	
						Marine n-3	1.06(0.76-1.48)	
						Total n-6	0.72(0.43-1.22)	
Hodge (2015) CC	Self-administered 121-item FFQ	Men & Women	CRC	395	3810	myristic acid	0.97(0.68-1.39)	Education, alcohol intake, smoking status, physical activity, total energy intake and stratified by: sex, ethnicity (Southern-European migrant vs. not) and family history of cancer.
						pentadecanoic acid	0.91(0.62-1.32)	
						PA	1.08(0.76-1.52)	
						SA	1.06(0.75-1.51)	
						SFA	0.93(0.66-1.32)	
						POA	1.04(0.74-1.47)	

						OA	1.27(0.89-1.81)	
						MUFAs	1.29(0.91-1.83)	
						Trans-FA	0.98(0.69-1.40)	
						LA	1.41(0.99-1.99)	
						AA	1.12(0.79-1.58)	
						n-6 PUFA	1.42(1.00-2.01)	
						ALA	1.09(0.77-1.53)	
						EPA	1.19(0.84-1.68)	
						DPA	1.00(0.71-1.42)	
						DHA	1.08(0.76-1.53)	
						n-3 PUFA	1.07(0.76-1.50)	
						Total PUFA	1.37(0.97-1.93)	
Shin (2020) CO	80-item validated FFQ	Women	CRC	334	48233	n-3 PUFA	1.21(0.88-1.67)	Body mass index (kg/m2; < 25, 25-30, ≥ 30, or missing), education (years; ≤ 10, 11-13, or > 13), smoking history (never or ever), and alcohol intake (g/day; < 5, 5-25, ≥ 25). Total caloric intake was adjusted by the residual method.
						ALA	1.17(0.86-1.59)	
						DPA	0.96(0.71-1.29)	
						EPA	0.92(0.69-1.24)	
						DHA	0.91(0.67-1.22)	
						n-6 PUFA	1.37(1.00-1.87)	
						LA	1.40(1.02-1.92)	
						AA	0.79(0.58-1.07)	
			Colon cancer	201	12056	n-3 PUFA	1.18(0.79-1.78)	
						ALA	0.96(0.65-1.41)	
						DPA	0.95(0.64-1.40)	
						EPA	1.11(0.76-1.62)	
						DHA	1.19(0.80-1.78)	
						n-6 PUFA	1.09(0.73-1.62)	

						LA	1.08(0.73-1.62)	
						AA	0.79(0.54-1.17)	
			Rectum cancer	133	12056	n-3 PUFA	1.25(0.74-2.12)	
						ALA	1.61(0.98-2.66)	
						DPA	0.97(0.61-1.55)	
						EPA	0.69(0.43-1.11)	
						DHA	0.62(0.39-0.98)	
						n-6 PUFA	1.98(1.17-3.32)	
						LA	2.11(1.25-3.59)	
						AA	0.79(0.49-1.29)	
Aglago (2020) CO	Diet questionnaire	Men & Women	CRC	6291	476160	EPA	0.86(0.78-0.95)	Body mass index, height, physical activity, smoking, education, and intakes of energy, alcohol, red and processed meat, fiber, and dairy products and stratified by age, sex, and center.
						DPA	0.83(0.75-0.92)	
						DHA	0.87(0.78-0.96)	
						n-6/n-3 PUFA	1.31(1.18-1.45)	
			Colon cancer	4080	476160	EPA	0.87(0.77-0.98)	
						DPA	0.83(0.73-0.94)	
						DHA	0.87(0.77-0.99)	
						n-6/n-3 PUFA	1.32(1.17-1.50)	
			Proximal Colon cancer	1877	476160	EPA	0.84(0.70-1.01)	
						DPA	0.85(0.71-1.03)	
						DHA	0.89(0.74-1.04)	
						n-6/n-3 PUFA	1.39(1.15-1.68)	
			Distal Colon cancer	1743	476160	EPA	0.94(0.78-1.13)	
						DPA	0.82(0.68-1.00)	
						DHA	0.89(0.74-1.08)	
						n-6/n-3 PUFA	1.14(0.94-1.39)	

			Rectum cancer	2094	476160	EPA	0.87(0.74-1.04)	
						DPA	0.84(0.71-1.01)	
						DHA	0.87(0.73-1.04)	
						n-6/n-3 PUFA	1.24(1.04-1.48)	
Aglago (2021) CO	FFQ	Men & Women	CRC	6162	450112	myristic acid	0.83(0.74-0.93)	BMI, height, physical activity (inactive, moderately inactive, moderately active, active), smoking, education, and dietary intakes of energy, red and processed meats, fiber, alcohol, and calcium and stratified by age, sex, and centre.
						pentadecanoic	1.05(0.95-1.17)	
						PA	0.81(0.70-0.93)	
						SA	0.88(0.76-1.01)	
						Total SFA	0.80(0.69-0.92)	
						POA	0.88(0.77-1.00)	
						OA	0.93(0.80-1.08)	
						Total MUFA	0.92(0.80-1.06)	
Nguyen (2021) CO	87-item validated semi-quantitative FFQ	Men & Women	CRC	876	59986	TFA	1.04(0.84-1.27)	Age, total energy intake (residual method), education levels, alcohol consumption (continuous drinks/day), family history of CRC, history of other diseases (cancer, myocardial infarction, stroke or diabetes mellitus), BMI, physical activity, fiber intake, calcium intake and was stratified income levels and cigarette smoking.
						SFA	0.92(0.74-1.14)	
						MUFA	0.95(0.79-1.16)	
						PUFA	1.18(0.95-1.46)	
						Total n-6PUFA	1.18(0.95-1.47)	
						LA	1.19(0.96-1.48)	
						AA	0.89(0.73-1.09)	
						Total n-3PUFA	1.09(0.87-1.36)	
						Marine PUFA	0.92(0.75-1.12)	
						ALA	1.15(0.92-1.43)	
						EPA	0.93(0.75-1.14)	
						DPA	0.99(0.81-1.21)	
						DHA	0.92(0.76-1.13)	
						n-6/n-3PUFA	1.11(0.91-1.36)	

			Caprylic acid	0.86(0.70-1.06)
			Capric acid	0.85(0.67-1.08)
			Myristic acid	0.90(0.70-1.15)
			Pentadecanoic	0.92(0.72-1.16)
			PA	1.00(0.81-1.24)
			SA	0.85(0.68-1.07)
			POA	0.93(0.76-1.13)
			OA	0.98(0.81-1.19)
			LA	1.19(0.96-1.48)
			ALA	1.15(0.92-1.43)
			AA	0.89(0.73-1.09)
			DPA	0.99(0.81-1.21)
			DHA	0.92(0.76-1.13)
Colon cancer	534	59986	TFA	0.98(0.75-1.28)
			SFA	0.86(0.65-1.12)
			MUFA	0.91(0.70-1.17)
			PUFA	1.10(0.84-1.45)
			Total n-6PUFA	1.12(0.85-1.47)
			Total n-3 PUFA	0.95(0.71-1.28)
			Marine PUFA	0.93(0.72-1.20)
			n-6/n-3 PUFA	1.19(0.92-1.54)
			Caprylic acid	0.80(0.61-1.05)
			Capric acid	0.93(0.68-1.27)
			Myristic acid	0.90(0.66-1.23)
			Pentadecanoic acid	0.93(0.68-1.26)
			PA	0.99(0.76-1.30)

			SA	0.87(0.65-1.16)
			POA	0.89(0.69-1.15)
			OA	0.98(0.76-1.26)
			LA	1.12(0.85-1.47)
			ALA	0.98(0.74-1.31)
			AA	0.93(0.72-1.20)
			DPA	1.03(0.80-1.34)
			DHA	0.95(0.73-1.22)
Rectum cancer	342	59986	TFA	1.14(0.82-1.57)
			SFA	1.03(0.73-1.44)
			MUFA	1.03(0.76-1.39)
			PUFA	1.31(0.94-1.84)
			Total n-6PUFA	1.30(0.92-1.83)
			Total n-3 PUFA	1.32(0.94-1.87)
			Marine PUFA	0.90(0.65-1.25)
			n-6/n-3 PUFA	0.98(0.71-1.36)
			Caprylic acid	0.95(0.69-1.32)
			Capric acid	0.75(0.51-1.10)
			Myristic acid	0.89(0.59-1.33)
			Pentadecanoic acid	0.90(0.61-1.32)
			PA	1.02(0.73-1.42)
			SA	0.82(0.57-1.18)
			POA	0.98(0.71-1.33)
			OA	0.99(0.73-1.34)
			LA	1.34(0.95-1.88)
			ALA	1.45(1.03-2.05)

						AA	0.84(0.61-1.16)	
						DPA	0.93(0.67-1.28)	
						DHA	0.89(0.65-1.23)	
Wan (2022) CO	Validated semi-quantitative FFQs	Men & Women	CRC	2726	111234	TFA	1.13(0.98-1.30)	Age , calendar time, family history of colorectal cancer (yes or no), prior lower gastrointestinal endoscopy (yes or no), pack-years of smoking before age 30 (0, 0–4, 4–10 and > 10), BMI (continuous), height (continuous), physical activity (< 3, 3–9, 9–18, 18–27 and > 27 METs/week), current multivitamin use (past use, currently continuously 1–5, 6–9, 10–15 and > 16 years of use), aspirin or NSAIDs use (past use, currently continuously 1–5, 6–9, 10–15 and > 16 years), hormone use (premenopausal/missing menopause, no history of use, current use or past use for women), total calorie (continuous), alcohol intake (0–4.9, 5–9.9, 10–14.9, 15–29.9 and > 30 g/d), energy-adjusted fiber (continuous), calcium (continuous), vitamin D (continuous), and total folate intake (continuous). All models, except total fat intake, mutually adjusted for various fat types.
						SFA	0.96(0.80-1.15)	
						MUFA	1.22(1.01-1.47)	
						PUFA	0.91(0.79-1.05)	
						Trans FA	0.96(0.82-1.12)	
						butyric acid	1.04(0.91-1.19)	
						caprylic acid	0.96(0.84-1.10)	
						capric acid	1.03(0.90-1.18)	
						myristic acid	1.00(0.87-1.15)	
						PA	1.09(0.94-1.26)	
						SA	1.12(0.96-1.30)	
						POA	1.02(0.89-1.18)	
						OA	1.08(0.91-1.28)	
						LA	0.99(0.87-1.12)	
						ALA	1.01(0.90-1.15)	
						AA	1.07(0.94-1.22)	
						EPA	1.07(0.94-1.23)	
						DPA	0.98(0.85-1.12)	
						DHA	0.99(0.87-1.14)	
						n-6 PUFA	0.98(0.87-1.11)	
						marine n-3	1.00(0.87-1.14)	
Ayako (2022)	33 food items FFQs	Men & Women	CRC	699	42,536	n-3 PUFA	1.11(0.84-1.46)	Age, sex, area, BMI, smoking status, alcohol consumption, walking, sports, education,
						LC n-3 PUFA	1.07(0.86-1.34)	

CO						ALA	0.97(0.69-1.36)	sedentary work, history of diabetes mellitus, family history of colorectal cancer, and quartiles of total energy, calcium, iron, saturated fatty acids, and fiber intake.
			Colon cancer	462	42,536	n-3 PUFA	1.11(0.79-1.56)	
						LC n-3 PUFA	1.09(0.83-1.43)	
						ALA	0.85(0.56-1.29)	
			Rectum cancer	237	42,536	n-3 PUFA	1.13(0.70-1.80)	
						LC n-3 PUFA	1.04(0.70-1.53)	
						ALA	1.24(0.70-2.19)	
Monireh (2022) CO	validated qualitatively Persian Cohort FFQ	Men & Women	CRC	865	4071	SFA	1.59(0.98-2.57)	Adjusted by province, age, SES, gender, BMI, tobacco use, opium use, aspirin use, physical activity, processed meat, fiber intake, calcium, and energy intake.
						Myristic acid	1.31(0.58-2.95)	
						Pentanoic acid	1.52(0.45-5.14)	
						Palmitic acid	1.01(0.40-2.52)	
						Heptanoic acid	0.33(0.14-0.79)	
						Stearic acid	0.91(0.39-2.12)	
						MUFA	0.96(0.57-1.64)	
						Palmitoleic acid	2.16(1.19-3.91)	
						Oleic acid	0.73(0.35-1.54)	
						PUFA	1.25(0.85-1.84)	
			Colon cancer	434	3640	SFA	1.18(0.95-1.46)	
						Myristic acid	1.35(0.96-1.89)	
						Pentanoic acid	1.82(1.20-2.77)	
						Palmitic acid	0.91(0.62-1.34)	
						Heptanoic acid	0.66(0.47-0.93)	
						Stearic acid	1.09(0.77-1.54)	
						MUFA	0.97(0.77-1.22)	
						Palmitoleic acid	1.22(0.95-1.56)	
						Oleic acid	0.70(0.52-0.95)	

						PUFA	1.09(0.92-1.29)	
			Rectum cancer	404	3610	SFA	1.10(0.89-1.36)	
						Myristic acid	0.91(0.65-1.27)	
						Pentanoic acid	0.93(0.55-1.56)	
						Palmitic acid	1.02(0.70-1.49)	
						Heptanoic acid	0.79(0.54-1.15)	
						Stearic acid	0.84(0.59-1.19)	
						MUFA	1.00(0.79-1.26)	
						Palmitoleic acid	1.18(0.92-1.51)	
						Oleic acid	1.09(0.80-1.48)	
						PUFA	1.03(0.87-1.22)	
Monireh (2022) CO	validated qualitative Persian Cohort food frequency questionnaire (FFQ)	Men & Women	CRC	865	4071	Trans-FA	1.10(1.01-1.19)	Adjusted by province, age, socioeconomic status, gender, aspirin intake, body mass index, tobacco, opium use, physical activity, processed meat, fiber intake, calcium, energy intake.
						Total industrial trans-FA	1.17(1.02-1.34)	
						elaidic acid	1.43(1.19-1.73)	
						Total ruminant trans-FA	0.85(0.73-0.89)	
						conjugated linoleic acid (CLAs)	0.93(0.75-1.15)	
			Colon cancer	434	3640	Trans-FA	1.10(0.99-1.22)	
						Total industrial trans-FA	1.28(1.07-1.54)	
						elaidic acid	1.58(1.24-2.02)	
						Total ruminant trans-FA	0.80(0.67-0.97)	
						conjugated linoleic	0.83(0.63-1.11)	

			acid (CLAs)	
Rectum cancer	404	3610	Trans-FA<br>Total industrial trans-FA<br>elaidic acid<br>Total ruminant trans-FA<br>conjugated linoleic acid (CLAs)	1.10(0.99-1.23)<br>1.07(0.88-1.31)<br>1.40(1.07-1.83)<br>0.86(0.70-1.05)<br>0.98(0.72-1.34)

[1]Abbreviations: AA: arachidonic acid; ALA: alpha linolenic acid; DHA: docosahexaenoic acid; DPA: docosapentaenoic acid; EPA: eicosapentaenoic acid; FA: fatty acids; LA: linoleic acid; MUFA: monounsaturated fatty acids'; OA: oleic acid; PA: palmitic acid; POA: palmitoleic acid; PUFA: polyunsaturated fatty acids; SA: stearic acid.

[2]CC: case-control study; CO: cohort study

[3]CRC: colorectal cancer.

Supplementary Table S5. Characteristics of the included studies in the meta-analysis.

**Author (year); study type**	**Measurement(blood)**	**Gender**	**Outcome types**	**Cases(n)**	**Participants(n)**	**Fatty acids**	**Adjusted RR or OR or HR (95% CI) (highest vs lowest intake)**	**Adjustment for confounding**
Hall (2007) CC	Gas-liquid chromatography	Men & Women	CRC	178	460	LC n-3 PUFAs	0.57(027-1.19)	Body mass index, multivitamin use, history of diabetes, random assignment to aspirin or placebo, vigorous exercise, alcohol intake, quartile of red meat intake, quartile of monounsaturated fatty acids and trans-unsaturated fatty acids.
						EPA	0.60(0.29-1.23)	
						DHA	0.69(0.39-1.23)	
						n-6 PUFAs	0.63(0.34-1.17)	
Kojima (2005) NCC	Gas chromatography	Men	CRC	83	324	SFA	1.71(0.66-4.47)	Family history of colorectal cancer in first-degree relatives, body mass index, education, smoking and alcohol drinking history, green leafy vegetable intake, and physical exercise.
						MUFA	2.05(0.86-4.89)	
						n-3PUFA	0.24(0.08-0.76)	
						n-6PUFA	0.69(0.30-1.61)	
						n-6/ n-3 ratio	2.05(0.78-5.40)	
						ALA	0.39(0.16-0.91)	
						EPA	0.44(0.18-1.08)	
						DPA	0.30(0.11-0.80)	
						DHA	0.23(0.07-0.76)	
						LA	0.57(0.24-0.38)	
						AA	1.16(0.49-2.75)	
		Women	CRC	86	326	SFA	0.59(0.23-1.52)	
						MUFA	0.83(0.36-1.92)	
						n-3PUFA	0.85(0.38-1.91)	

						n-6PUFA	1.15(0.48-2.75)	
						n-6/ n-3 ratio	1.08(0.50-2.36)	
						ALA	2.19(0.87-5.47)	
						EPA	0.83(0.39-1.80)	
						DPA	0.64(0.30-1.39)	
						DHA	0.80(0.33-1.93)	
						LA	1.88(0.78-4.52)	
						AA	0.65(0.30-1.44)	
Zhang (2015) CC	Gas chromatography	Men & Women	CRC	100	162	Total n-3 PUFA	4.06(1.57-10.51)	Potential confounding factors, including age, sex, smoke, alcohol drinking, body mass index (BMI), total cholesterol, LDL cholesterol, HDL cholesterol, triglycerides, total energy, protein, fat and carbohydrate intake.
						ALA	1.23(0.50-3.02)	
						EPA	2.05(0.79-5.31)	
						DPA	0.96(0.39-2.35)	
						DHA	3.79(1.45-9.87)	
						Total n-6 PUFA	8.15(2.77-23.92)	
						LA	3.67(1.42-9.47)	
						AA	2.71(1.05-7.00)	
						n-6/n-3	0.52(0.21-1.31)	
Butler (2017) CC	Gas chromatography	Men & Women	Colon cancer	211	422	PA	0.41(0.20-0.83)	Adjusted for body mass index (<20, 20–24, 24–28, ≥28 kg/m2), smoking (never, light, heavy), education level (none, primary, ≥secondary), alcohol use (none, <7, ≥7 drinks/week), weekly physical activity (yes, no), history of diabetes (yes, no), and use of nonsteroidal anti-inflammatory drugs (NSAIDs) (yes, no).
						SA	0.55(0.30-0.99)	
						POA	0.51(0.28-0.95)	
						OA	0.39(0.20-0.76)	
						ALA	0.41(0.23-0.73)	
						EPA	0.93(0.54-1.62)	
						DHA	1.11(0.59-2.07)	
						LA	0.43(0.23-0.82)	
						AA	1.52(0.82-2.81)	

			Rectum cancer	139	278	PA	1.50(0.65-3.44)	
						SA	1.72(0.79-3.75)	
						POA	1.10(0.51-2.39)	
						OA	1.77(0.80-3.91)	
						ALA	1.70(0.84-3.43)	
						EPA	0.89(0.42-1.91)	
						DHA	0.72(0.32-1.66)	
						LA	1.15(0.51-2.58)	
						AA	1.44(0.63-3.27)	
Aglago （2021） CO	Gas chromatography	Men & Women	Colon cancer	433	866	myristic acid	0.51(0.32-0.83)	BMI (continuous), height(continuous), physical activity (inactive, moderately inactive, moderately active, active), smoking, education (none, primary, technical and professional, secondary, higher education), and dietary intakes of energy (continuous), red and processed meats (continuous), fiber (continuous), alcohol (continuous), and calcium (continuous).
						pentadecanoic	0.63(0.37-1.06)	
						palmitic acid	0.88(0.54-1.43)	
						stearic acid	1.63(1.00-2.64)	
						Total SFA	1.04(0.64-1.68)	
						palmitoleic	0.75(0.44-1.27)	
						acid	0.91(0.57-1.45)	
						oleic acid	0.97(0.61-1.54)	
						MUFA		
Aglago (2020) CC	Gas chromatography	Men & Women	CRC	461	922	EPA	0.89(0.59-1.35)	Body mass index, height, physical activity, smoking, education, and intakes of energy, alcohol, red and processed meat, fiber, and dairy products.
						DPA	1.18(0.73-1.91)	
						DHA	1.19(0.76-1.85)	
						n-3 LC-PUFA	0.94(0.61-1.44)	
						n-6: n-3PUFA	0.87(0.55-1.36)	
			Colon cancer	455	916	EPA	0.89(0.59-1.35)	
						DPA	1.18(0.73-1.92)	
						DHA	1.19(0.76-1.86)	

						n-3 LC-PUFA	0.94(0.61-1.44)	
						n-6:n-3PUFA	0.86(0.55-1.35)	
			Proximal	185	646	EPA	0.79(0.41-1.50)	
			Colon			DPA	0.99(0.44-2.22)	
			cancer			DHA	0.75(0.37-1.53)	
						n-3 LC-PUFA	0.55(0.27-1.11)	
						n-6:n-3PUFA	0.74(0.33-1.64)	
			Distal	222	683	EPA	1.00(0.50-2.00)	
			Colon			DPA	1.75(0.83-3.68)	
			cancer			DHA	1.92(0.93-3.94)	
						n-3 LC-PUFA	1.54(0.77-3.08)	
						n-6:n-3PUFA	0.69(0.35-1.36)	
Hodge	gas-liquid	Men & Women	CRC	395	3810	myristic acid	1.05(0.74-1.50)	Education, alcohol intake, smoking status,
(2015)	chromatograph					pentadecanoic	0.58(0.39-0.85)	physical activity, total energy intake and
CASE-COHORT						palmitic acid	1.53(1.05-2.22)	stratified by: sex, ethnicity (Southern-
						stearic acid	1.00(0.70-1.42)	European
						SFA	1.53(1.08-2.16)	migrant vs. not) and family history of cancer.
						palmitoleic	1.02(0.72-1.45)	
						acid	1.05(0.73-1.52)	
						oleic acid	0.91(0.63-1.31)	
						MUFAs	0.87(0.58-1.31)	
						Trans fatty acid	1.42(1.00-2.01)	
						linoleic acid	0.71(0.50-1.02)	
						arachidonic	1.06(0.75-1.49)	
						acid	0.96(0.69-1.33)	
						PUFA n-6	0.84(0.60-1.19)	

ALA	0.71(0.49-1.01)
EPA	0.71(0.51-0.99)
DPA	0.77(0.55-1.08)
DHA	1.07(0.75-1.52)
PUFA n-3	
Total PUFA	

[1]Abbreviations: AA: arachidonic acid; ALA: alpha linolenic acid; DHA: docosahexaenoic acid; DPA: docosapentaenoic acid; EPA: eicosapentaenoic acid; FA: fatty acids; LA: linoleic acid; MUFA: monounsaturated fatty acids'; OA: oleic acid; PA: palmitic acid; POA: palmitoleic acid; PUFA: polyunsaturated fatty acids; SA: stearic acid.

[2]CC: case-control study; CO: cohort study

[3]CRC: colorectal cancer.

Supplementary Table S6. Characteristics of the included Mendelian Randomization studies of the plasma fatty acids and colorectal cancer.

Reference	Exposure (FAs)	Instrumental Variables (IV)	Main MR analysis	Outcome Sample		Results (per SD-unit change in exposure)		MR evidence supporting a causal effect	Evidence category
				Source	Case/control	OR (95% CI)	P-value		
Wilson, 2017	Arachidic acid	rs680379	Wald ratio	CCFR1, CCFR2, COIN, FINLAND, UK1, Scotland1 and VQ58	9,254/18,386	0.92(0.61,1.39)	0.7	No	Non-evaluable
	PA	rs2391388	Wald ratio			0.97(0.78,1.21)	0.82	No	
	SA	rs6675668, rs11119805, rs102275	IVW			1.16(1.01,1.35)	0.04	Yes	
	DHA	rs2236212	Wald ratio			1.32(0.94,1.87)	0.11	No	
	DPA	rs780094, rs3734398, rs174547	IVW			1.58(0.99,2.52)	0.06	No	
	EPA	rs3798713	Wald ratio			0.39(0.13,1.21)	0.1	No	
	AA	rs174547, rs16966952	IVW			1.05(1.02,1.07)	1.7E-04	Yes	
	DGLA	rs174547, rs16966952	IVW			0.91(0.83,1.00)	0.06	No	
	LA	rs10740118, rs174547, rs16966952	IVW			0.95(0.93,0.98)	3.7E-04	Yes	
	POA	rs780093, rs6722456, rs603424, rs11190604, rs102275	IVW			0.77(0.65,0.92)	3.9E-03	Yes	
	OA	rs102275	Wald ratio			0.36(0.15,0.84)	0.018	Yes	

Liyanage, 2019	LA	rs10740118, rs16966952, rs174547	IVW	UK Biobank cohort	13,696/270,342	0.95(0.93,0.97)	9.6E-05	Yes	Suggestive evidence
	AA	rs16966952, rs174547	IVW			1.05(1.03,1.07)	2.0E-05	Yes	
	ALA	rs174547	Wald ratio			0.89(0.78,1.02)	0.098	No	
	DPA	rs174547, rs3734398	IVW			1.10(1.01,1.19)	0.034	Yes	
	EPA	rs3798713, rs174538	IVW			1.06(0.91,1.22)	0.455	No	
	DHA	rs2236212	Wald ratio			1.07(0.84,1.36)	0.583	No	
Khankari, 2020	LA	rs10740118, rs174547, rs2727270, rs16966952	IVW	GECCO	11,016/13,732	0.96(0.93,0.98)	5.2E-04	Yes	Robust evidence
	AA	rs174547, rs16966952	IVW			1.06(1.03,1.08)	3.3E-05	Yes	
	ALA	rs174547	Wald ratio			0.95(0.92,0.97)	5.4E-05	Yes	
	EPA	rs3798713, rs174538	IVW			1.04(1.01,1.07)	2.5E-03	Yes	
	DPA	rs780094, rs3734398, rs174547	IVW			1.03(1.01,1.06)	1.2E-02	Yes	
	DHA	rs2236212	Wald ratio			1.01(0.98,1.03)	0.53	No	
Larsson, 2021	AA	rs174547, rs16966952	IVW	UK Biobank, FinnGen, BBJ	14,121/691,910	1.08(1.05,1.11)	6.3E-08	Yes	Suggestive evidence
	EPA	rs3798713, rs174538	IVW			1.20(1.11,1.31)	<0.001	Yes	
	DHA	rs2236212	Wald ratio			1.00(0.81,1.22)	0.970	No	

[1]Abbreviations: AA: arachidonic acid; ALA: alpha linolenic acid; DHA: docosahexaenoic acid; DPA: docosapentaenoic acid; DGLA: Dihomo-g-linolenic acid;

EPA: eicosapentaenoic acid; FAs: fatty acids; LA: linoleic acid; OA: oleic acid; PA: palmitic acid; POA: palmitoleic acid; SA: stearic acid.

[2]BBJ: BioBank Japan; CCFR1: Colon Cancer Family Registry; CCFR2: Colon Cancer Family Registry; COIN: COIN trial; FINLAND: Finnish Colorectal Cancer Predisposition Study; Scotland1: COGS (Colorectal Cancer Susceptibility; GECCO: the Genetics and Epidemiology of Colorectal Cancer Consortium; UK1: CORGI (colorectal Tumour Gene Identification Consortium).

[3]CRC: colorectal cancer.
